# Nanosized Li_2_S‐Loaded Polar Porous Carbon Nanofibers as Self‐Supporting Electrodes in Anode‐Free Lithium–Sulfur Batteries

**DOI:** 10.1002/advs.202516575

**Published:** 2025-12-08

**Authors:** Ping Feng, Qingping Wu, Yaolin Xu, Liqiang Lu, Tianle Zheng, Tonghui Xu, Wen Xu, Daniel Höche, Zdravko Kochovski, Yan Lu

**Affiliations:** ^1^ Institute of Electrochemical Energy Storage Helmholtz‐Zentrum Berlin für Materialien und Energie 14109 Berlin Germany; ^2^ Institute for Technical Chemistry and Environmental Chemistry Friedrich‐Schiller‐Universität Jena 07743 Jena Germany; ^3^ Chongqing Institute of Green and Intelligent Technology Chinese Academy of Sciences 400714 Chongqing China; ^4^ School of Natural Sciences Technical University of Munich (TUM) James‐Franck‐Str. 1 85748 Garching Germany; ^5^ Department of Chemistry College of Sciences Shanghai University Shanghai 200444 P. R. China; ^6^ Institute of Surface Science Helmholtz‐Zentrum Hereon Max‐Planck‐Str. 1 21502 Geesthacht Germany; ^7^ Helmholtz Institute for Polymers in Energy Applications Jena (HIPOLE Jena) 07743 Jena Germany

**Keywords:** anode‐free, electrospinning, lithium dendrite, lithium–sulfur batteries, polar host

## Abstract

Anode‐free lithium–sulfur (Li–S) batteries with Li_2_S as the cathode offer a promising alternative to improve practical energy density but suffer from sluggish redox kinetics on the cathode side and chaotic Li plating/stripping process on the copper current collectors. In this work, phosphorus‐doped porous carbon nanofibers (P‐CNFs) are served both as self‐standing hosts for Li_2_S cathode and 3D current collectors for Li deposition. In the cathode, nanoscale Li_2_S particles (less than 10 nm in size) are in situ synthesized via carbon thermal reduction of lithium sulfate which is confined within the brush layer of anionic spherical polyelectrolyte brushes. The incorporation of Li_2_S nanoparticles within the void of P‐CNFs (Li_2_S@P‐CNFs) imparts unimpeded electron/ion transport at the polar carbon matrix interface, thus enhancing the Li_2_S conversion reaction kinetics and mitigating the shuttling effect of polysulfides during cycling. Moreover, the lithiophilic P‐CNFs skeleton with interconnected macropores effectively homogenizes Li plating behavior, resulting in smooth and compact deposition morphology. As a result, the Li_2_S@P‐CNFs||P‐CNFs full cell delivers a low‐capacity decay of 0.051% cycle^−1^ for 1000 cycles at 1 C. This work gives a unique strategy for the practicalization of anode‐free Li–S batteries, with the potential to extend to other battery systems.

## Graphical abstract

Anode‐free Li–S cells are realized by coupling nano‐Li_2_S embedded in P‐doped porous carbon nanofibers (Li_2_S@P‐CNFs) with lithium‐free P‐CNF current collectors. Polar P‐CNFs accelerate Li_2_S conversion and suppress shuttling, while their lithiophilic, macroporous network homogenizes Li plating for dendrite‐free deposits. The full cell sustains 1000 cycles at 1 C with only 0.051% capacity fade per cycle.

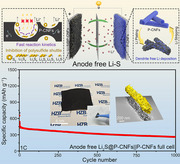

## 1 Introduction

Lithium–sulfur (Li–S) batteries are considered as one of the most promising next‐generation rechargeable batteries because of their high theoretical energy density (up to 2600 Wh kg^−1^) arising from the high specific capacity of sulfur cathode (1672 mAh g^−1^) and Li anode (3860 mAh g^−1^).^[^ ^1^, ^2^, ^3^ ^]^ However, a large excess of Li metal with an unrealistic Li to S capacity ratio of 20 or even higher significantly diminishes the practical energy densities of Li–S batteries.^[^ ^4^, ^5^ ^]^ Anode‐free battery designs, which use fully lithiated cathodes as the Li source and bare current collectors on the anode side, offer a compelling alternative.^[^ ^6^, ^7^, ^8^ ^]^ These configurations not only enhance energy density and reduce costs but also eliminate the need for direct Li anode integration during battery assembly.^[^ ^9^ ^]^

Lithium sulfide (Li_2_S), with a high Li content of 66.7 at%, is a promising Li‐rich cathode material for anode‐free Li–S batteries. It eliminates the need for problematic Li metal anodes while maintaining high energy density. In addition, Li_2_S offers a high theoretical capacity of 1166 mAh g^−1^ through a multi‐electron redox reaction.^[^ ^10^ ^]^ As a fully lithiated cathode, Li_2_S does not experience volume expansion during the lithiation–delithiation process because it is already at its maximum volume state, making it suitable for manufacturing thick and high‐loading electrodes.^[^ ^11^ ^]^ In addition, Li_2_S can withstand high temperatures due to its excellent thermal stability, with a melting point of 938 °C,^[^ ^12^ ^]^ allowing various high‐temperature (>500 °C) options for the rational synthesis of nanostructured Li_2_S cathodes, which is, however, impossible for sulfur due to its thermal instability. Unfortunately, Li_2_S is electrochemically inactive and typically suffers from high initial activation overpotential (≈3.5 V vs Li/Li^+^) and sluggish reaction kinetics due to its poor electrical conductivity and low solubility in organic electrolytes.^[^ ^13^ ^]^ Meanwhile, similar to sulfur cathodes, a range of soluble lithium polysulfide intermediates (LiPSs, Li_2_S*_x_*, 4 ≤ *x* ≤ 8) are produced during charging and discharging.^[^ ^14^, ^15^, ^16^ ^]^ These intermediates can cause the crossover of LiPSs, known as the “shuttle effect,” leading to reduced Coulombic efficiency and fast capacity decay. Furthermore, Li_2_S is costly ($10.6−$12.9 kg^−1^), which limits its economic advantage compared to sulfur.^[^ ^17^ ^]^

To address these issues and improve the electrochemical performance of Li_2_S cathodes, various strategies have been proposed, including utilizing nanosize effects,^[^ ^18^, ^19^ ^]^ incorporating electrocatalysts or redox mediators,^[^ ^20^, ^21^, ^22^ ^]^ as well as engineering electrolytes.^[^ ^23^ ^]^ Embedding nanosized Li_2_S particles in the conductive matrix, such as carbon nanotubes,^[^ ^10^, ^24^ ^]^ carbon nanofibers,^[^ ^17^, ^25^, ^26^, ^27^ ^]^ porous carbon,^[^ ^28^ ^]^ and (reduced) graphene oxide,^[^ ^18^, ^19^, ^29^, ^30^ ^]^ facilitates electron and ion transport, reducing the initial overpotential down to 3.0 V (vs Li/Li^+^) and extending the cycle life to several hundred of cycles.^[^ ^31^, ^32^, ^33^ ^]^ However, these designs are limited by the weak physical interactions between the carbon scaffolds and LiPSs, leading to unsatisfactory cyclability.^[^ ^34^ ^]^ Introducing metal‐based electrocatalysts with higher polysulfide adsorption ability, including metal sulfides,^[^ ^35^, ^36^, ^37^ ^]^ metal phosphides,^[^ ^38^ ^]^ and MXenes,^[^ ^39^ ^]^ is promising, but these metal compounds have limited conductivity, slowing electron and Li^+^ transport.^[^ ^34^, ^40^ ^]^ In this regard, introducing a small amount of polar atoms (such as N, O, or P) into the carbon matrix is highly desirable. This modification not only changes the polarity of carbon, enhancing its physical adsorption ability, but also creates active sites for chemical adsorption and catalytic LiPSs conversion without reducing surface area or pore volume.^[^ ^41^, ^42^, ^43^ ^]^

On the anode side of anode‐free Li–S batteries, bare copper (Cu) foil without Li is often employed as the anode, offering a promising pathway towards achieving ultrahigh energy density.^[^ ^44^ ^]^ However, the chaotic Li plating/stripping processes on the lithiophobic Cu foil often result in dendritic or mossy Li growth.^[^ ^45^, ^46^ ^]^ Such an irregular Li deposition, together with uncontrollable side reactions between fresh Li and electrolytes, causes the accumulation of dead Li and the depletion of electrolytes.^[^ ^47^ ^]^ To address these challenges, two primary stabilization strategies have been proposed: (1) utilizing high surface area hosts to reduce local current density; and (2) interfacial engineering to develop artificial solid electrolyte interphase (SEI) layers on the Li surface.^[^ ^48^ ^]^ Porous carbon nanofibers embedded with lithiophilic sites are ideal host materials for Li anode, offering advantages such as confined space for controlled Li deposition and stabilized SEI layers.^[^ ^49^ ^]^ These porous structures can accommodate volume changes during Li cycling, while inner lithiophilic sites, such as heteroatom dopants (e.g., N, O, and P), reduce nucleation overpotential and guide uniform Li deposition.^[^ ^50^ ^]^ The selective deposition of Li in the porous carbon nanofiber mitigates dendrite formation and minimizes side reactions between Li and electrolyte, thereby improving Coulombic efficiency and cyclic stability.

Herein, we present a novel anode‐free Li–S full cell configuration based on Li_2_S, designed to extend the lifespan of these batteries through an electrospinning strategy. The proposed design features dual‐functional P‐doped porous carbon nanofibers (P‐CNFs), prepared by electrospinning followed by calcination, working both as freestanding hosts for Li_2_S cathodes and 3D current collectors for Li anodes. The P‐CNFs exhibit dual functionality: they serve not only as an electrocatalyst for Li_2_S cathodes but also as a freestanding host for the Li anode, enabling the construction of anode‐free Li–S batteries. This dual role effectively addresses two major challenges in anode‐free batteries: the sluggish kinetics of the Li_2_S redox reactions and the unstable Li plating behavior on the anode side. Utilizing electrostatic interactions between the negatively charged brushes in the spherical polyelectrolyte brush (SPB) and the Li^+^ in the lithium sulfate, Li_2_S nanoparticles were generated in situ in the porous P‐CNFs (Li_2_S@P‐CNFs) matrix by using lithium sulfate as a cheap precursor and SPB as a soft template. This not only helps to load the active materials homogeneously in the cathode but also optimizes the reaction interface, creating shorter electron/ion transport paths. In addition, P doping was derived from the phosphoric acid in the precursor solution, which improved the electrochemical reaction kinetics. On the anode side, P‐CNFs with lithiophilic sites modulate the homogeneous deposition of high‐capacity Li and maintain an efficient and dendrite‐free Li plating/stripping process as confirmed by SEM and in situ optical measurement. Finite element simulations revealed a homogenous electrical field distribution in P‐CNFs that can mitigate the concentration gradient of Li^+^, thus alleviating the space charge layer shielding effect, thereby suppressing Li dendrite growth. Even at the maximum deposition capacity of 49 mAh cm^−2^, the plated nanofibers maintained a smooth and compact morphology. The resulting state‐of‐the‐art anode‐free Li_2_S@P‐CNFs||P‐CNFs full cell delivered an initial specific discharge capacity of 654.6 mAh g^−1^ at 1 C with a low‐capacity decay of 0.051% cycle^−1^ for 1000 cycles (calculated from the second cycle). The Li_2_S@P‐CNFs‖P‐CNFs full cell exhibits the lowest capacity decay rate among previously reported conventional anode‐free Li‐S batteries based on Li_2_S cathodes. Although there are few reported studies in the literature that have developed electrode materials capable of serving as both sulfur cathodes and Li anodes,^[^ ^51^, ^52^ ^]^ this is the first demonstration of a P‐doped carbon nanofiber film, which can be used as a bifunctional host in anode‐free Li_2_S batteries without any excess Li, achieving both enhanced redox kinetics and stabilized Li plating/stripping behavior, highlighting the novelty and potential impact of our approach. The success of our design provides a unique strategy based on electrospinning for advancing anode‐free Li–S batteries, with potential applicability to other battery systems.

## 2 Results and Discussion

**Figure 1:**
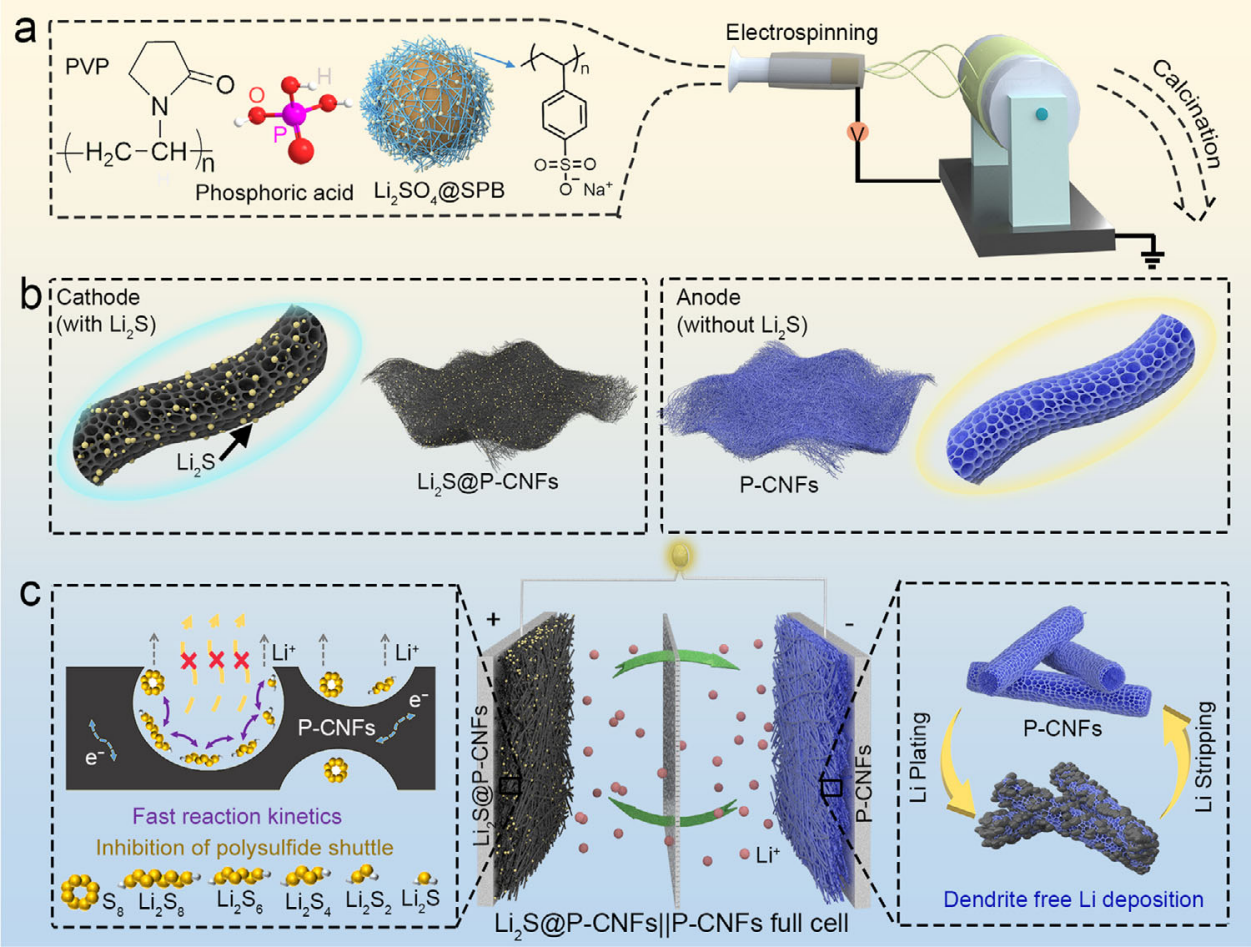
a,b) Synthesis scheme for the Li_2_S@P‐CNFs cathode and the P‐CNFs current collectors, and c) schematic illustration of the anode‐free Li_2_S@P‐CNFs||P‐CNFs full cell configuration and the role played by P‐CNFs in sulfur chemistry and Li deposition.

The synthesis strategy for the Li_2_S@P‐CNFs electrode is schematically illustrated in **Figure** **1**. SPB templates with an anionic sodium poly(styrene sulfonate) brush layer were first synthesized according to previous report.^[^ ^53^, ^54^ ^]^ Transmission electron microscopy (TEM) image of the SPB (Figure S1, Supporting Information) shows homogeneous spherical particles with a diameter of ≈100 nm. No brushes were detected on the surface of SPB due to the “dry effect” during TEM sample preparation. The hydrodynamic diameter of SPB is 478.3 ± 136.3 nm and the thickness of the brush layer is around 195.8 nm according to dynamic light scattering (DLS) measurements (Figure S2, Supporting Information). Commercial polyvinylpyrrolidone (PVP) polymer and lithium sulfate (Li_2_SO_4_) were dissolved in an aqueous SPB dispersion to prepare the precursor solution for electrospinning. A 1% volume fraction of phosphoric acid (PA) solution was added as a phosphorus source.^[^ ^53^, ^54^ ^]^ Notably, the anionic brush layer of SPB plays a crucial role in maintaining the uniform dispersion of the SPB solution. As shown in Figure S2a (Supporting Information), the polystyrene solution aggregates after the addition of 5 wt% Li_2_SO_4_, while the SPB solution remains colloidally stable under the same conditions. The DLS results show that the average hydrodynamic diameter of the SPB decreases from 478.3 ± 136.3 nm to 283.7 ± 74.2 nm after adding 5 wt% Li_2_SO_4_ (Figure S2b, Supporting Information), due to the strong electrostatic interaction between the anionic sodium poly(styrene sulfonate) brush layer and Li_2_SO_4_. The confinement of Li_2_SO_4_ in the brush layer prevents its agglomeration during the subsequent electrospinning and calcination processes, facilitating the homogeneous dispersion of Li_2_SO_4_ across the nanofibers, and ultimately achieving ultrasmall Li_2_S nanoparticles.^[^ ^36^ ^]^ The SPB template can be synthesized on a large scale with good reproducibility, as has been demonstrated in our previous studies. Moreover, Li_2_SO_4_ is an inexpensive precursor, and PVP is a widely available commercial polymer. Therefore, our synthesis method is not only scalable but also economically viable.^[^ ^16^ ^]^

**Figure 2:**
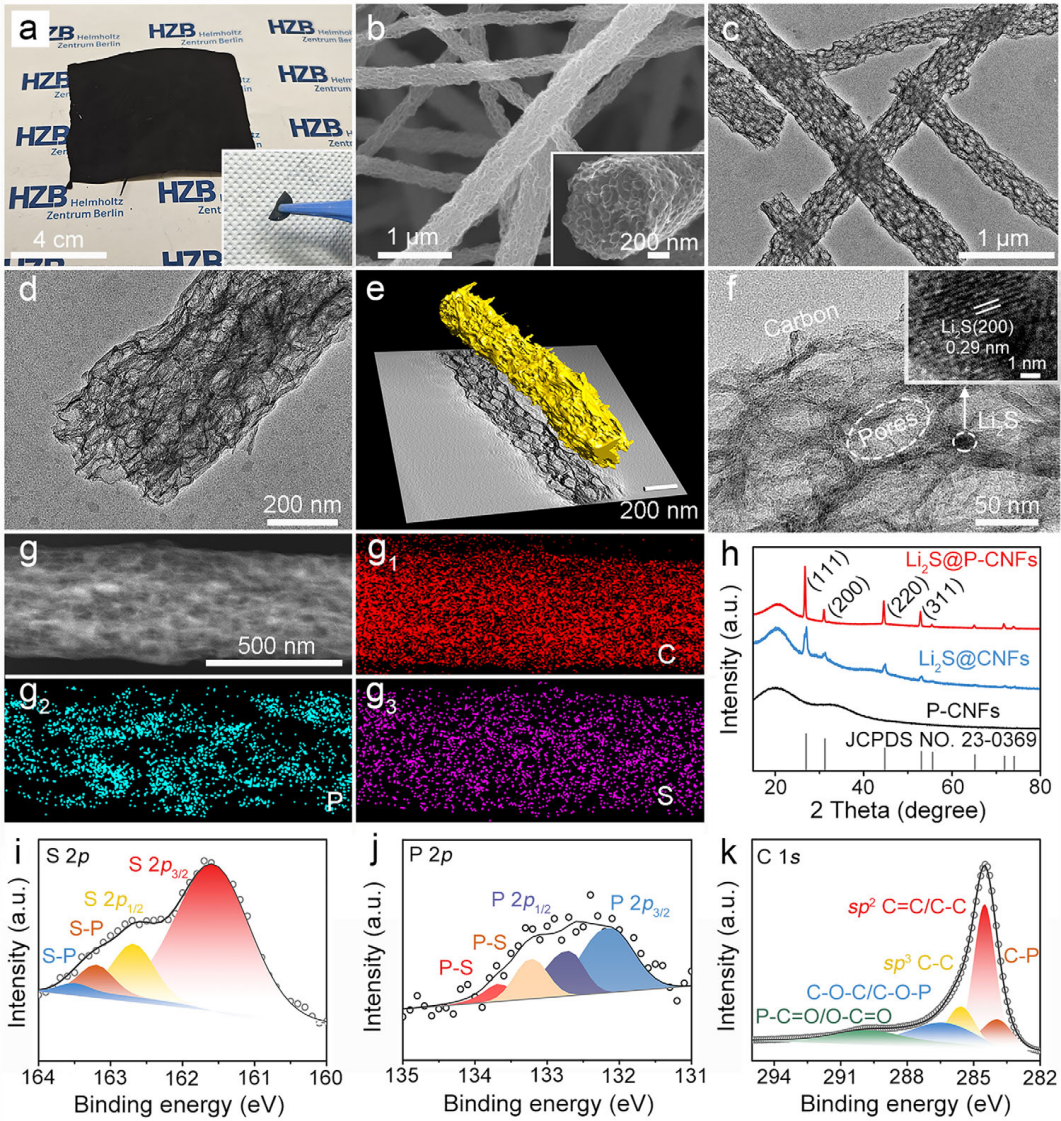
a) Digital image of the as‐prepared Li_2_S@P‐CNFs. b) SEM image, and c,d) TEM images of the Li_2_S@P‐CNFs. e) 3D volume rendering from a TEM tomographic reconstruction of a single P‐CNFs nanofiber, shown over a central XY slice from the reconstruction. f) HRTEM images of the Li_2_S@P‐CNFs. g) TEM image and the corresponding C, P, and S elemental mapping images of the Li_2_S@P‐CNFs. h) XRD patterns of the Li_2_S@P‐CNFs, Li_2_S@CNFs, and P‐CNFs. XPS spectra of the i) S 2*p*, j) P 2*p*, and k) C 1*s* of the Li_2_S@P‐CNFs.

The mixture solution of SPB with PVP, Li_2_SO_4_, and PA was electrospun into freestanding nonwoven fabrics under ambient conditions (Figure S3a, Supporting Information). Subsequently, the freestanding nonwoven fabrics underwent pre‐oxidation at 150 °C for 2 h in the air to induce polymer cross‐linking.^[^ ^56^ ^]^ The pre‐oxidation process could enhance the carbon yield during the following calcination processes under Ar and facilitate the formation of freestanding fabrics (Figure S3b, Supporting Information). Finally, the prepared fabrics were calcined at 900 °C for 2 h under an Ar atmosphere (Figure S3c, Supporting Information). During this process, Li_2_SO_4_ was in situ reduced to Li_2_S by the surrounding carbon (Li_2_SO_4_ + 2C → Li_2_S + 2CO_2_). Meanwhile, PA was pyrolyzed into phosphides or phosphorus oxides, which are doped into the carbon structure, resulting in the final product of nanosized Li_2_S embedded in P‐doped porous carbon nanofibers (Li_2_S@P‐CNFs).^[^ ^35^ ^]^ The Li_2_S@P‐CNFs membranes demonstrate excellent flexibility after calcination from the digital images (**Figure** **2**a), and can be cut into self‐supporting electrodes that serve directly as the cathode for Li–S batteries.

The detailed morphology and structure of the Li_2_S@P‐CNFs were examined using scanning electron microscopy (SEM) and TEM. The Li_2_S@P‐CNFs retain the nanofiber structure of the polymer nanofibers, and the average diameter of Li_2_S@P‐CNFs is 340 ± 69 nm, as shown in Figure S4 (Supporting Information). Notably, numerous macropores are evenly distributed throughout the Li_2_S@P‐CNFs, resulting from the low carbon yield of SPB nanospheres during calcination (Figure 2b).^[^ ^53^, ^54^ ^]^ TEM images of the Li_2_S@P‐CNFs further confirm the formation of porous nanofibers and the homogeneous distribution of interconnected macropores with a size of about 50 nm, throughout the P‐CNFs (Figure 2c,d). The inner structural details of the P‐CNFs were studied by reconstructed electron tomography (ET), as shown in Figure 2e and Figure S5 (Supporting Information). Together with the tomograms provided in Video S1 (Supporting Information), 3D interconnected macropores can be clearly observed in the highly porous P‐CNFs nanofibers. The uniform distribution of interconnected macropores provides robustness to nanofibers, making them suitable as freestanding electrodes.^[^ ^57^ ^]^ In addition, the highly porous P‐CNFs facilitate unimpeded electron/ion transport at the polar carbon matrix interface.^[^ ^58^ ^]^

High‐resolution TEM (HRTEM) images of the Li_2_S@P‐CNFs reveal the uniform decoration of Li_2_S nanoparticles, less than 10 nm in size, within the P‐CNFs (Figure 2f). The uniform decoration of ultra‐small Li_2_S nanoparticles is primarily attributed to the strong electrostatic interaction between the anionic sodium poly(styrene sulfonate) brush layer and Li_2_SO_4_. This confinement ensures the homogeneous distribution of Li_2_SO_4_ across the nanofibers, ultimately leading to the formation of ultrasmall Li_2_S nanoparticles.^[^ ^36^ ^]^ The inverse FFT image's line profile confirms a *d*‐spacing of 2.9 Å for Li_2_S nanoparticles, corresponding to the (200) plane of Li_2_S (Figure S6, Supporting Information), while the surrounding carbon matrix remains disordered.^[^ ^17^ ^]^ The even distribution of nanosized Li_2_S reduces the initial activation barrier, shortens ionic diffusion distances, and improves redox reaction kinetics, thereby enhancing electrode performance.^[^ ^28^ ^]^ Energy‐dispersive X−ray spectroscopy (EDS) mapping, shown in Figure 2g, examines the distribution of different elements in the Li_2_S@P‐CNFs. It clearly indicates that the S element is evenly distributed throughout the P‐CNFs, confirming the homogeneous embedding of nanosized Li_2_S. The presence of phosphorus is also confirmed by EDS, signifying successful doping of phosphorus. Elemental analysis reveals a phosphorus doping content of 7.4 wt%. The phosphorus doping content was also measured by inductively coupled plasma mass spectrometry (ICP‐MS) analysis. The results indicate a phosphorus doping content of 7.9 wt%, which is consistent with the values obtained from elemental analysis. Nitrogen doping was also detected in the ICP‐MS results, with a doping content of 1.7 wt%, which is significantly lower than that of phosphorus. Due to its low concentration, the effect of nitrogen doping on the electrochemical performance is considered negligible.

The specific surface area and pore structure of Li_2_S@P‐CNFs were characterized using nitrogen adsorption–desorption isotherms. As shown in Figure S7 (Supporting Information), the nitrogen adsorption–desorption isotherms of Li_2_S@P‐CNFs display a type IV isotherm with a specific surface area of 81.7 m^2^ g^−1^, and the pore size distribution is primarily centered at 3.6 nm. The crystallinity of Li_2_S@P‐CNFs was examined by X‐ray powder diffraction (XRD), as shown in Figure 2h. Two control samples were prepared using the same method as Li_2_S@P‐CNFs, but one without Li_2_SO_4_ to obtain pure P‐doped CNFs (P‐CNFs) and the other without PA to obtain Li_2_S nanoparticles embedded in non‐doped CNFs (Li_2_S@CNFs). P‐CNFs and Li_2_S@CNFs feature numerous evenly distributed macropores throughout the nanofibers, resembling the porous structure of Li_2_S@P‐CNFs, as shown in the TEM images in Figure S8 (Supporting Information). In the diffraction pattern of P‐CNFs, two broad peaks located around 20° and 32° can be attributed to amorphous carbon.^[^ ^59^ ^]^ After introducing Li_2_SO_4_, new peaks located at 26.7°, 31.0°, 44.6°, and 52.9° appeared in the diffraction pattern of Li_2_S@CNFs, which correspond to the (111), (200), (220), and (311) planes of cubic Li_2_S (JCPDS No. 23‐0369), respectively.^[^ ^17^ ^]^ There are no impurities detected in the diffraction pattern of Li_2_S@CNFs, indicating that all Li_2_SO_4_ was converted to Li_2_S during the high‐temperature carbon thermal reduction. The peaks in the Li_2_S@P‐CNFs sample remain unchanged compared to those of Li_2_S@CNFs, suggesting that PA does not disturb the formation of Li_2_S but results in elemental P doping into the carbon. The Li_2_S content in Li_2_S@P‐CNFs was determined using thermogravimetric analysis (TGA), as shown in Figure S9 (Supporting Information). Due to the air sensitivity of Li_2_S, the TGA measurement started with the polymer nanofibers prepared with Li_2_SO_4_@SPB. The sample was heated from room temperature to 900 °C under an Ar flow at a rate of 5 °C min^−1^. During this process, PVP pyrolyzed to carbon, SPB burned out at 300−500 °C, and Li_2_SO_4_ was reduced to Li_2_S by carbon at 500−800 °C. After cooling to room temperature under Ar and changing the gas flow to dry air, the sample was further heated to 800 °C at 5 °C min^−1^. During this stage, Li_2_S and carbon were oxidized to Li_2_SO_4_ (Figure S10, Supporting Information) and CO_2_, respectively. The amount of Li_2_S in Li_2_S@P‐CNFs was calculated to be 55 wt% based on the residues.^[^ ^28^ ^]^

The chemical composition of Li_2_S@P‐CNFs was further analyzed using X‐ray photoelectron spectroscopy (XPS). The Li 1*s* spectrum of Li_2_S@P‐CNFs displays two broad peaks at 55.0 and 54.4 eV (Figure S11, Supporting Information), corresponding to the Li─S and Li─P bonds, respectively.^[^ ^28^ ^]^ The S 2*p* spectrum of Li_2_S@P‐CNFs shows peaks at 161.6 and 162.8 eV corresponding to the S 2*p*_3/2_ and S 2*p*_1/2_ of the S─Li bond (Figure 2i), respectively. Those XPS results indicate the successful synthesis of Li_2_S@P‐CNFs, consistent with the XRD results. Moreover, peaks located at 163.2 and 163.5 eV are observed in the deconvoluted S 2*p* spectrum of Li_2_S@P‐CNFs, corresponding to the S─P bond.^[^ ^28^ ^]^ The S─P bond has also been observed in the deconvoluted P 2*p* spectrum of Li_2_S@P‐CNFs (Figure 2j), centered at 133.7 and 133.2 eV.^[^ ^28^ ^]^ Meanwhile, the peaks centered at 132.1 (2*p*_3/2_) and 132.7 eV (2*p*_1/2_) in the deconvoluted P 2*p* spectrum of Li_2_S@P‐CNFs can be assigned to the phosphorus doping in carbon, while no such peak is present in the P 2*p* spectrum of the reference sample Li_2_S@CNFs (Figure S12, Supporting Information). The P─S bonding plays a critical role in the following ways:^[^ ^60^, ^61^ ^]^ (i) The formation of P─S bonds effectively immobilizes soluble lithium polysulfides (Li_2_S*_n_*, 4 ≤ *n* ≤ 8) by anchoring them onto the host material. This chemical confinement hinders the dissolution and diffusion of polysulfides into the electrolyte, thereby mitigating the shuttle effect that typically leads to capacity fading and poor coulombic efficiency. (ii) The P‐doping modifies the electronic structure of the carbon host, introducing localized electron‐rich sites that can enhance the adsorption and conversion of polysulfides. This not only stabilizes intermediate species but also accelerates the redox reactions during charge/discharge processes, contributing to improved reaction kinetics and better rate capability. (iii) Phosphorus atoms, due to their larger atomic radius and electron‐donating properties, can increase the electrical conductivity of the carbon matrix. Moreover, the formation of stable P─S bonds contributes to maintaining the integrity of the sulfur cathode during cycling. Previous reports have shown that P─S bonds lead to the formation of lithium polysulfidophosphate (Li*_x_* PS*_y_*) species, which act as fast Li^+^ conductors and facilitate the activation of bulk Li_2_S particles.^[^ ^28^ ^]^ The C 1*s* spectrum of Li_2_S@P‐CNFs shows peaks at 289.8, 286.5, 285.6, 284.5, and 283.9 eV (Figure 2k), corresponding to C═O, C─O, C─C, C═C, and C─P bonds, respectively,^[^ ^62^ ^]^ confirming the successful doping of phosphorus. Raman spectroscopy of Li_2_S@P‐CNFs, Li_2_S@CNFs, and CNFs is presented in Figure S13 (Supporting Information). The spectra for all three samples exhibit two main peaks at 1350.0 and 1591.8 cm^−1^, corresponding to the D band and G band of carbon, respectively.^[^ ^63^ ^]^ The D to G band intensity ratio, indicative of the defect level in carbon, is slightly higher in Li_2_S@P‐CNFs (1.1) compared to that of Li_2_S@CNFs (1.0) and CNFs (1.0), which is attributed to phosphorus doping.

**Figure 3:**
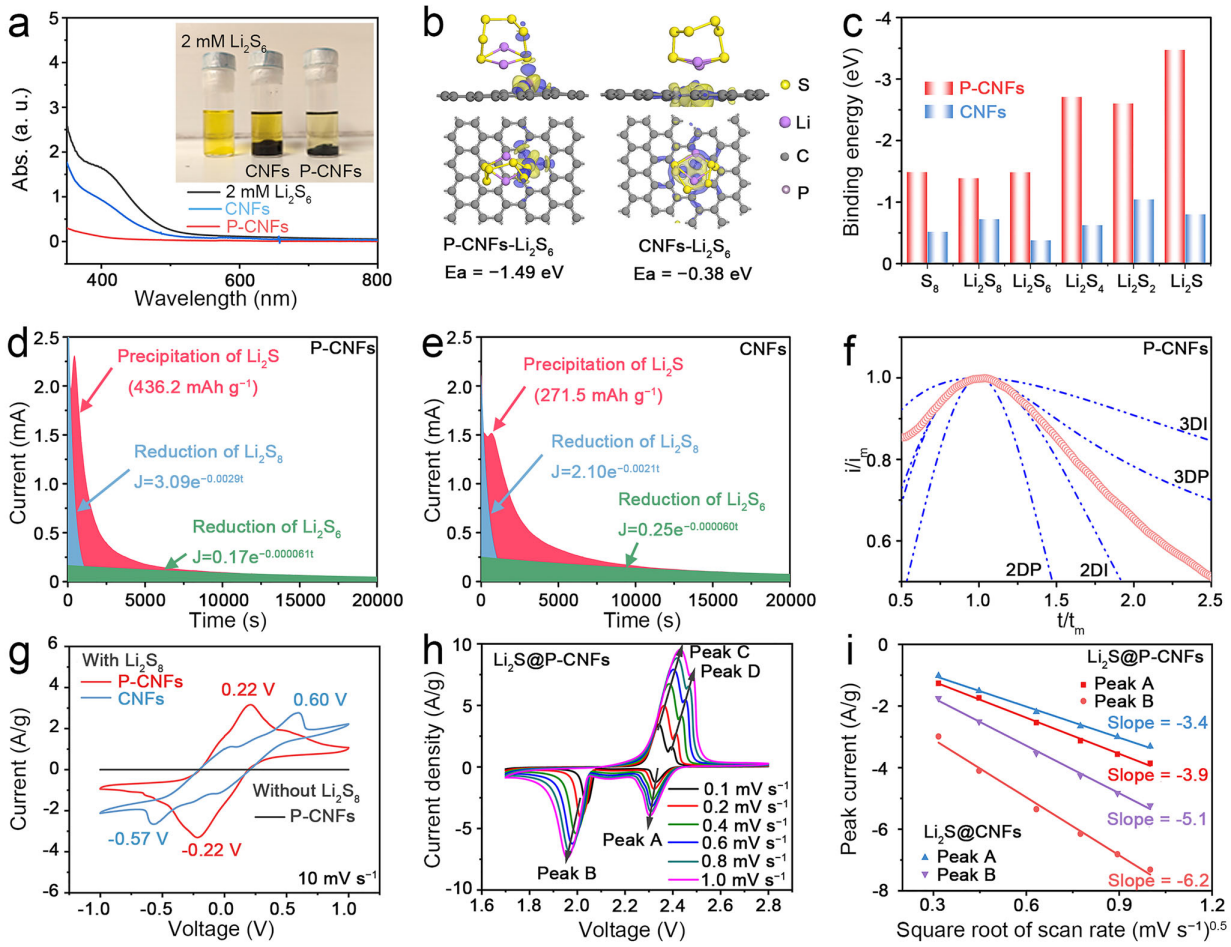
a) UV–vis absorption spectra of the Li_2_S_6_ electrolyte after mixing with 20 mg of P‐CNFs and CNFs for 3 h. Inset: Digital image of the Li_2_S_6_ electrolyte after mixing with 20 mg of P‐CNFs and CNFs for 3 h. b) Optimized configurations for the binding of Li_2_S_6_ to P‐CNFs and CNFs. c) Binding energy (in eV) of the Li_2_S_x_ cluster adsorbed on P‐CNFs and CNFs. Fitting of the current–time curves for constant voltage discharge at 2.05 V on d) P‐CNFs and e) CNFs. f) Corresponding dimensionless transients of P‐CNFs compared with theoretical growth models. (*t*: time, *t*_m_: time needed to reach the maximum current, *i*: current, *i*_m_: maximum current.) g) CV curves of the Li_2_S_8_ symmetric cell with P‐CNFs and CNFs as electrodes at a scan rate of 10 mV s^−1^ in the voltage range of −1.0 to 1.0 V. h) CV curves of the coin cells with the Li_2_S@P‐CNFs electrode after activated Li_2_S to S in the voltage range of 1.7–2.8 V at various scan rates of 0.1, 0.2, 0.4, 0.6, 0.8, and 1.0 mV s^−1^. i) The plot of CV peak current of the peak A (S_8_→Li_2_S_4_) and peak B (Li_2_S_4_→Li_2_S) versus the square root of scan rate.

Heteroatom P has been reported to significantly change the electronic structure and chemical activity of the carbon framework, enhancing the adsorption capability and catalytic activity of LiPSs and improving the cycling stability in Li–S batteries.^[^ ^64^ ^]^ The catalytic activity of P‐CNFs toward Li–S redox reactions was evaluated to study the effect of heteroatom P on the electrochemical behavior. The catalysis process includes three key steps: (1) adsorption of reactants onto the catalyst surface, (2) diffusion of adsorbed species to active sites, and (3) desorption of products after the catalysis.^[^ ^38^ ^]^ Thus, the adsorption capability of the LiPSs, a critical preliminary step in catalysis, was first evaluated through a static adsorption experiment using a 2 × 10^−3^ m Li_2_S_6_ solution in 1,3‐dioxolane (DOL)/1,2‐dimethoxyethane (DME) (V_DOL_:V_DME_ = 1:1), as illustrated in **Figure** **3**a.^[^ ^65^ ^]^ Equal mass of P‐CNFs and CNFs (20 mg of each) was introduced into 4 mL of the 2 × 10^−3^ m Li_2_S_6_ solution. After standing for 3 h, the yellow color of the Li_2_S_6_ solution mixed with P‐CNFs and CNFs showed a noticeable decrease compared to the initial solution, indicating the adsorption of Li_2_S_6_ by both materials. The supernatant Li_2_S_6_ solutions were then subjected to ultraviolet–visible (UV–vis) absorption spectroscopy, as depicted in Figure 3a. The UV–vis absorption spectra of 2 × 10^−3^ m Li_2_S_6_ solution present a notable absorption intensity in the wavelength range of 350–500 nm. After introducing different host materials and standing for 3 h, the 2 × 10^−3^ m Li_2_S_6_ solution treated with P‐CNFs exhibited lower absorption intensity within the wavelength range of 350–500 nm compared to that of CNFs, indicating their superior LiPSs adsorption capability due to P doping. The enhanced LiPSs adsorption capabilities of P‐CNFs were further validated by density functional theory (DFT) calculations. Figure 3b and Figure S14 (Supporting Information) display the optimized adsorption models for representative LiPSs species (Li_2_S, Li_2_S_2_, Li_2_S_4_, Li_2_S_6_, Li_2_S_8_, and S_8_) with P‐CNFs and CNFs. The binding energies of P‐CNFs for different LiPSs species are calculated to be −3.5, −2.6, −2.7, −1.5, −1.4, and −1.5 eV for Li_2_S, Li_2_S_2_, Li_2_S_4_, Li_2_S_6_, Li_2_S_8_, and S_8_, respectively (Figure 3c). Those binding energies are much lower than that of CNFs (−0.8, −1.0, −0.6, −0.4, −0.7, and −0.5 eV for Li_2_S, Li_2_S_2_, Li_2_S_4_, Li_2_S_6_, Li_2_S_8_, and S_8_, respectively), which is consistent with the Li_2_S_6_ adsorption experiment shown in Figure 3a. These results suggest that P‐CNFs play a significant role in immobilizing the LiPSs due to polar–polar interactions,^[^ ^66^ ^]^ aligning with the LiPSs adsorption tests and DFT calculation results presented above.

The impact of host materials on sulfur redox reactions is significantly influenced by their interactions with both liquid‐phase and solid‐phase LiPSs. Particularly, the conversion from high‐order to low‐order LiPSs, ultimately precipitating into Li_2_S is crucial since this process accounts for 75% of theoretical capacity and encompasses the most kinetically sluggish solid‐phase conversion.^[^ ^67^, ^68^, ^69^ ^]^ Given the significantly stronger binding of Li_2_S on P‐CNFs (−3.5 eV for P‐CNFs compared to −0.8 eV for CNFs), it is likely that polar P‐CNFs nanofibers can serve as a sulfiphilic platform to effectively guide the uniform deposition of Li_2_S.^[^ ^70^ ^]^ Here, we conducted potentiostatic nucleation and growth of Li_2_S to investigate the electrochemical deposition from liquid LiPSs to solid Li_2_S on both P‐CNFs and CNFs nanofibers. The P‐CNFs‐based electrode achieves a higher current of 2.31 mA after 420 s (Figure 3d), compared to the CNFs‐based electrode, which reaches 1.52 mA after 655 s (Figure 3e). Moreover, the P‐CNFs‐based electrode exhibits a higher Li_2_S precipitation capacity of 436.2 mAh g^−1^ compared to 271.5 mAh g^−1^ for the CNFs‐based electrode, indicating rapid nucleation and deposition of Li_2_S on the P‐CNFs surface. The nucleation and deposition behavior of P‐CNFs and CNFs were further analyzed using four classic electrochemical deposition models: Scharifker–Hills, and Bewick, Fleischman, and Thirsk models. The 2D instantaneous (2DI) and progressive (2DP) nucleation suggest a two‐dimensional mechanism, where growth is primarily governed by lattice incorporation. The 3D progressive (3DP) and instantaneous (3DI) nucleation indicate that ion diffusion dictates the growth rate of a three‐dimensional hemispherical nucleus.^[^ ^71^ ^]^ As shown in Figure 3f and Figure S15 (Supporting Information), the growth pattern of Li_2_S in the P‐CNFs electrode exhibits a mixed behavior of 2DI and 3DP nucleation, indicating that Li_2_S growth is controlled by both lattice bonding and ion diffusion. In contrast, the growth pattern of Li_2_S in the CNFs electrode aligns with 3DP nucleation, where Li_2_S growth is primarily controlled by ion diffusion. These observations are strong evidence that the polar P‐CNFs not only effectively immobilize liquid‐phase LiPSs, but also process the capability for their facile conversion into low‐order LiPSs.

Having separately analyzed the interactions and conversions of high‐order and low‐order LiPSs with the host materials, it is essential to conduct a comprehensive electrochemical evaluation of P‐CNFs and CNFs in half cells, where all these processes occur simultaneously. In this regard, cyclic voltammetry (CV) was employed with symmetric cells (Figure 3g), where P‐CNFs or CNFs served as both working and counter electrodes, and Li_2_S_8_ was added to the electrolyte. It is evident that the current peak, derived from the redox behavior of the P‐CNFs/Li_2_S_6_ interface, occurs significantly earlier in both cathodic and anodic peaks compared to that of the CNFs sample. Moreover, the current density of the Li_2_S_8_ symmetric cell with P‐doped CNFs is higher than that of the CNFs‐based cell, confirming the faster reaction kinetics of polysulfide conversion on the P‐CNFs surface. This finding aligns with previous DFT calculations on the reaction free energy of LiPSs with phosphorus‐doped carbon materials, which demonstrated that phosphorus‐doped carbon materials significantly lower the energy barriers for the redox reaction of LiPSs compared to those of undoped carbon materials, thereby facilitating enhanced reaction kinetics.^[^ ^60^ ^]^ The evaluation of the catalytic activity on the LiPSs conversion highlights the P‐CNFs as the most efficient host materials for the Li–S batteries system compared to CNFs. The next step involves applying these host materials to Li–S cells to confirm their electrochemical performances. The electrochemical reversibility of the cells was first examined using CV measurements in a half‐cell configuration (Li metal works as the anode). To activate Li_2_S to S, the coin cells were initially oxidized to 3.5 V (Figure S16, Supporting Information) and then scanned within the voltage window of 1.9–2.8 V.^[^ ^39^ ^]^ As shown in Figure 3h and Figure S17a (Supporting Information), both CV curves of Li_2_S@P‐CNFs and Li_2_S@CNFs‐based electrodes exhibit two cathodic peaks and two anodic peaks. The first cathodic peak at 2.3 V (peak A) corresponds to the reduction of sulfur into long‐chain LiPSs, while the second cathodic peak at 2.0 V (peak B) corresponds to the further reduction into short‐chain LiPSs. The first anodic peak at 2.3 V (peak C) corresponds to the oxidation of short‐chain LiPSs into long‐chain LiPSs, and the second anodic peak at 2.4 V (peak D) corresponds to the further oxidation into elemental sulfur.^[^ ^72^ ^]^

To further understand the sulfur conversion kinetics in half‐cells, the Li^+^ diffusion was measured. Figure 3i and Figure S17b (Supporting Information) display the plot of CV peak current for the cathodic and anodic peaks versus the square root of the scan rate. All peaks exhibit a linear relationship with the square root of the scan rates, indicating a diffusion‐limited process.^[^ ^73^ ^]^ Consequently, Li‐ion diffusion coefficient can be determined using the Randles–Sevcik Equation (1):

(1) $$I_{p}=\left(2.69\times 10^{5}\right)n^{1.5}A{D_{\mathrm{Li}}}^{0.5}C_{\mathrm{Li}}v^{0.5}$$

where *I*_p_, *n*, *A*, *D*_Li_, *C*_Li_, and *v* represent the peak current, the number of electrons transferred, the surface area of the electrode, the Li^+^ diffusion coefficient, the concentration of Li^+^ ions in the cathodes, and the scan rate, respectively. The slope values of the curves (*I*_p_/*v* ^0.5^) for cathodic peaks A and B and anodic peaks C and D for Li_2_S@P‐CNFs and Li_2_S@CNFs are calculated and summarized in Table S1 (Supporting Information). Compared to Li_2_S@CNFs, improved Li^+^ diffusion has been approved in the Li_2_S@P‐CNFs‐based electrodes, indicated by the higher slope of the *I*_p_ versus *v* ^0.5^ curves. As presented in Table S1 (Supporting Information), the calculated 𝐷_𝐿𝑖_ values of Li_2_S@P‐CNFs are 1.1 × 10^−8^, 2.9 × 10^−8^, 5.6 × 10^−8^, and 5.6 × 10^−8^ for peaks A, B, C, and D, respectively. These values are notably higher than those obtained for Li_2_S@CNFs, which are 8.7 × 10^−9^, 1.9 × 10^−8^, 3.2 × 10^−8^, and 4.0 × 10^−8^ cm^2^ s^−1^, respectively. The enhanced diffusion coefficients observed for Li_2_S@P‐CNFs can be attributed to the strong interactions between Li^+^ and P active sites, which facilitate more efficient Li⁺ transport within the sulfur host matrix. Consequently, the polar P‐CNFs with accelerated Li^+^ diffusion facilitate charge transfer between P‐CNFs nanofiber hosts and LiPSs guests, thereby accelerating the charge/mass diffusion and the overall conversion reaction.

A series of catalytic activity evaluations has clarified the enhanced conversion kinetics driven by the P‐CNFs for the Li–S system. To confirm this in the Li–S half cells, electrochemical performances of the Li_2_S@P‐CNFs and Li_2_S@CNFs electrodes were evaluated. In Figure S18a (Supporting Information), the pre‐charge curve of the Li_2_S@P‐CNFs and Li_2_S@CNFs cathodes with Li_2_S loading of ≈1.0 mg cm^−2^ is illustrated. Li_2_S@P‐CNFs and Li_2_S@CNFs cathodes exhibit an initial charge capacity of 1000.6 and 727.2 mAh g^−1^, respectively, when charged to 3.5 V at 0.05 C (1 C = 1166 mA g^−1^ of Li_2_S). All the capacities are calculated based on Li_2_S. Upon the initial charge, the Li_2_S@P‐CNFs cathode requires a low voltage of ≈2.4 V to activate solid‐state Li_2_S, while the Li_2_S@CNFs cathode needs ≈2.6 V for activation. The activation potential barrier of the Li_2_S@P‐CNFs cathode has also been compared with other reported Li_2_S‐based cathodes with similar Li_2_S loading, as shown in Figure S18b (Supporting Information), which shows the lowest activation voltage of the Li_2_S@P‐CNFs cathode. Moreover, a reference sample of bulk Li_2_S loaded on P‐CNFs has been prepared (denoted as Li_2_S/P‐CNFs) and the electrochemical performance of Li_2_S/P‐CNFs has been measured under the same conditions (Figure S19, Supporting Information). Due to the high activation barrier of the bulk Li_2_S, only a small amount of Li_2_S can be activated, and the following discharge capacity is as low as 30 mAh g^−1^. This comparison highlights the advantages of the nanosized Li_2_S embedded in porous P‐CNFs synchronously with P doping in facilitating the activation of Li_2_S and improving active material utilization.^[^ ^39^ ^]^

The cycling performance of Li_2_S@P‐CNFs and Li_2_S@CNFs after activation was evaluated at 0.2 C, as depicted in Figure S20a (Supporting Information), to investigate their impact on the electrochemical performance of Li–S batteries. The Li_2_S@P‐CNFs electrode demonstrated an initial specific discharge capacity of 726.8 mAh g^−1^ at 0.2 C, maintaining 535.8 mAh g^−1^ after 100 cycles, resulting in a capacity retention rate of 73.7% (0.26% cycle^−1^). In comparison, Li_2_S@CNFs electrodes exhibited an initial specific discharge capacity of 434.4 mAh g^−1^, which decreased to 243.2 mAh g^−1^ after 100 cycles under the same conditions, corresponding to a capacity retention rate of 56.0% (0.44% cycle^−1^). The galvanostatic charge–discharge (GCD) curves of the Li_2_S@P‐CNFs and Li_2_S@CNFs electrodes are presented in Figure S20b (Supporting Information), displaying two discharge plateaus at ≈2.4 and 2.1 V and two charge plateaus at ≈2.3 and 2.4 V, respectively, similar to the CV results. Notably, the charge–discharge potential gap (Δ*E*) is 160 mV for the Li_2_S@P‐CNFs electrode and 220 mV for the Li_2_S@CNFs electrode, indicating the improved reaction kinetics of the Li_2_S@P‐CNFs electrode. The discharge cutoff voltage was set to 1.9 V to prevent irreversible decomposition of LiNO_3_, as demonstrated in Figure S21 (Supporting Information).^[^ ^74^ ^]^ The electrochemical impedance spectroscopy (EIS) of the Li_2_S@P‐CNFs and Li_2_S@CNFs electrodes before cycling is shown in Figure S22 (Supporting Information). EIS shows a semicircle in the high‐frequency region denoting the charge‐transfer process at the interface, while a linear section in the low‐frequency region represents lithium diffusion within the electrode.^[^ ^51^ ^]^ As shown in Figure S22 (Supporting Information), the resistance of the Li_2_S@P‐CNFs was only 26.5 Ω, lower than that of Li_2_S@CNFs (84.5 Ω). The smaller resistance of the Li_2_S@P‐CNFs electrode indicates faster electronic mobility and improved electrochemical kinetics due to the phosphorus doping. By simply tuning the amount of H_3_PO_4_ solution added, we obtained the optimal level of phosphorus doping. The influence of the phosphorus doping level on cycling performance is provided in the Experimental Section and Figure S23 (Supporting Information).

The rate performance of the Li_2_S@P‐CNFs and Li_2_S@CNFs electrodes and the corresponding GCD curves are shown in Figure S24 (Supporting Information). The Li_2_S@P‐CNFs electrode exhibits specific discharge capacities of 504.2, 437.5, 378.2, 337.5, and 302.4 mAh g^−1^ at 0.1, 0.2, 0.5, 1.0, and 2.0 C, respectively. A reversible specific discharge capacity of 412.7 mAh g^−1^ was achieved when the current density was shifted back to 0.2 C, demonstrating its good rate capability. In contrast, the specific discharge capacities of the Li_2_S@CNFs electrode maintained at 398.2, 354.4, 304.1, 266.8, and 230.8 mAh g^−1^ at current densities of 0.1, 0.2, 0.5, 1.0, and 2.0 C, respectively. When the current density shifted back to 0.2 C, the specific discharge capacity was only maintained at 344.4 mAh g^−1^. These performance results correlate well with the trend from the series of catalyst performance tests, confirming that P‐CNFs can work efficiently as electrocatalysts for sulfur redox reactions.

**Figure 4:**
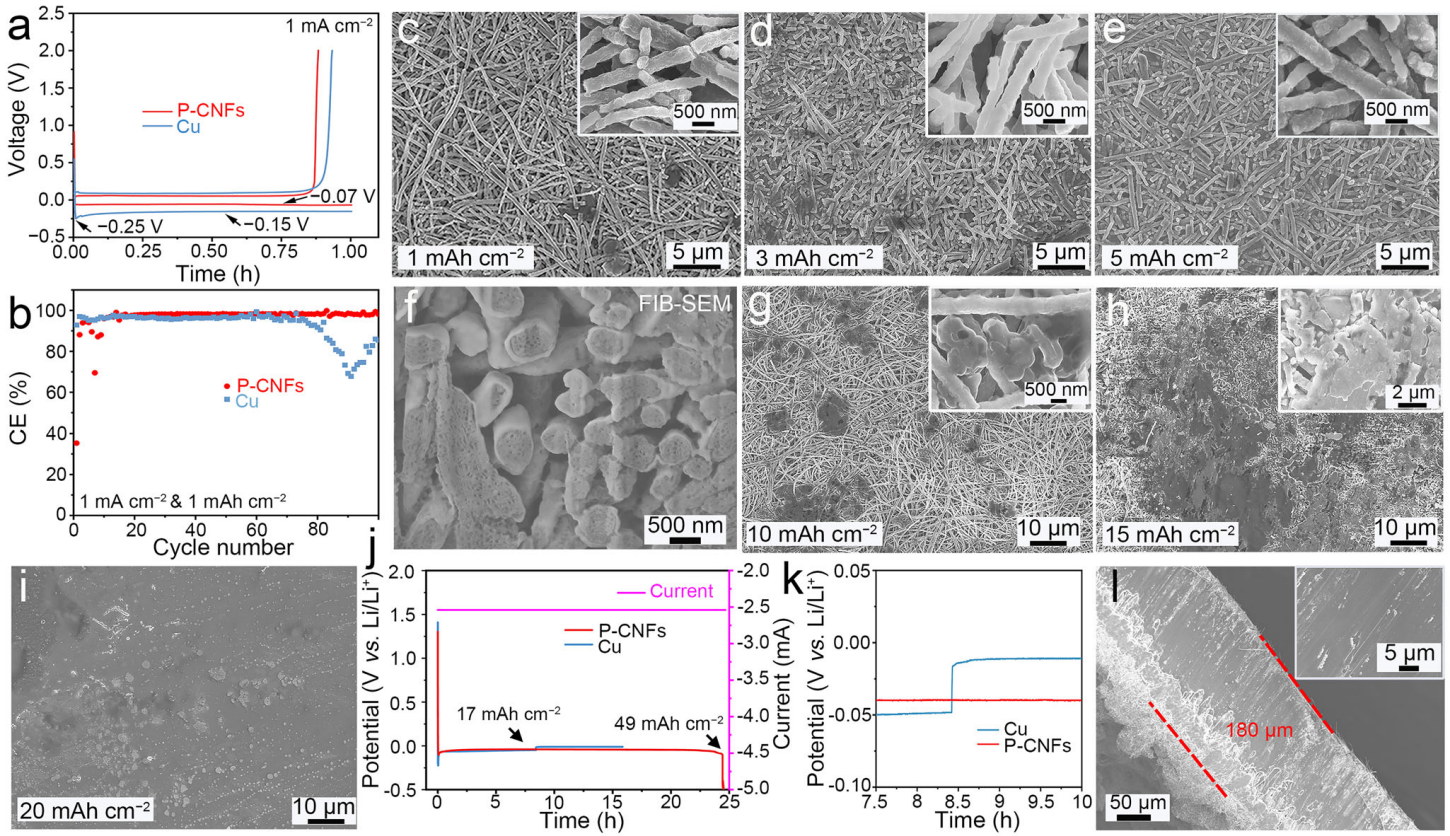
a) Voltage profiles and b) Coulombic efficiencies of Li plating and stripping on P‐CNFs and Cu current collectors at a current density of 1 mA cm^−2^. SEM images of the P‐CNFs current collector with c) 1 mAh cm^−2^, d) 3 mAh cm^−2^, e) 5 mAh cm^−2^, g) 10 mAh cm^−2^, h) 15 mAh cm^−2^, and i) 20 mAh cm^−2^ of Li deposition. f) The FIB/SEM image of the P‐CNFs current collector with 5 mAh cm^−2^ of Li deposition. j,k) The discharge curve of the Li||P‐CNFs and Li||Cu cells at a current density of 2 mA cm^−2^. l) Cross‐section SEM image of the P‐CNFs current collector after 49 mAh cm^−2^ Li deposition. Inset: Surface SEM image of the P‐CNFs current collector after 49 mAh cm^−2^ Li deposition.

It is noteworthy that the P‐CNFs exhibit dual functionality: they can serve not only as the electrocatalyst for Li_2_S cathodes but also as the free‐standing host for Li anode to construct anode‐free Li–S batteries. The interconnected porous nanofiber network not only provides abundant catalytic sites for polysulfide conversion in the cathode but also ensures mechanical robustness and uniform Li‐ion flux distribution in the anode. More importantly, the phosphorus doping introduces lithiophilic sites, which help to guide uniform Li deposition and suppress dendrite growth, thereby mitigating excessive SEI accumulation despite the porous structure. In particular, the efficiency of Li plating/stripping plays a critical role in determining the overall performance of anode‐free Li–S batteries. Porous carbon nanofibers, with their 3D conductive networks, have been widely reported to be used as the current collector on the anode to mitigate Li dendrite growth.^[^ ^75^ ^]^ As a result, they are highly desirable as the current collectors in anode‐free Li–S batteries, offering significant potential for improving cycling stability. The Li plating and stripping behaviors were initially explored on P‐CNFs and Cu current collectors (conventional current collectors for anode‐free Li–S batteries^[^ ^76^ ^]^) in half cells, where P‐CNFs or Cu current collectors served as the working electrode and metallic Li as the counter electrode. Before cycling, the half cells were preconditioned with 3 cycles at 0.1 mA cm^−2^ from 0.1 to 2.0 V to establish a relatively stable SEI on the current collectors (Figure S25, Supporting Information). The half‐cell underwent galvanostatic charge and discharge at a current density of 1 mA cm^−2^ and a capacity of 1 mAh cm^−2^ to assess the Coulombic efficiency of Li plating and stripping, which reflects the cyclic reversibility and stability of the cells. The charge–discharge curves of the Li||Cu and Li||P‐CNFs cells are depicted in **Figure** **4**a. During discharge, P‐CNFs current collectors exhibit a relatively flat voltage plateau, and the Li nucleation overpotential, defined as the voltage difference between the bottom of the voltage dip and the subsequent mass‐transfer plateau, is as low as 70 mV. In contrast, Li deposition on bare Cu current collectors initially shows a significant voltage dip (nucleation overpotential of 250 mV), which is negligible in the Li||P‐CNFs cell. This result highlights the superior lithiophilic nature of P‐CNFs, making them more favorable for Li deposition.^[^ ^77^ ^]^ The variations in Coulombic efficiency (the ratio of Li stripping to Li plating) for the Li||Cu and Li||P‐CNFs cells are plotted in Figure 4b. Li could deposit and strip on Cu current collectors for 70 cycles at a current density of 1 mA cm^−2^ with an average Coulombic efficiency higher than 85%. However, the Coulombic efficiency of Cu current collectors rapidly declined after 70 cycles due to Li dendritic growth and repetitive SEI formation/destruction involving electrolyte consumption. In contrast, during the initial ten cycles, which can be ascribed to the gradual activation and wetting of the porous carbon nanofiber host. After this activation process, it delivers stable Li plating/stripping for nearly 100 cycles with a high Coulombic efficiency (>97%). This enhanced cycling performance correlates with the minimal nucleation overpotential of the P‐CNFs plating during Li plating, promoting uniform Li⁺ migration and stable cycling.

To elucidate the reasons behind the enhanced Li plating/stripping efficiencies on P‐CNFs current collectors compared to Cu, ex situ SEM analysis was conducted alongside in situ characterizations. The micro‐morphology evolution of Li during plating on Cu and P‐CNFs current collectors at various areal capacities was investigated using ex situ SEM.^[^ ^50^ ^]^ As depicted in Figure S26 (Supporting Information), when the plating capacity was 1 mAh cm^−2^, the Cu current collectors surface exhibited mossy fiber‐like dendritic Li with diameters around 3 µm. Increasing the plating capacity to 3 and 5 mAh cm^−2^ resulted in more pronounced Li dendrites growing to diameters of 5 and 6 µm, respectively. The thicknesses of Li deposits on the Cu current collectors were ≈14, 20, and 60 µm at areal deposition capacities of 1, 3, and 5 mAh cm^−2^, respectively, much larger than the corresponding calculated theoretical values of 4.8, 14.4, and 24.2 µm (details in Equation S3, Supporting Information), due to the loosely stacked mossy fiber‐like Li dendrites.

In contrast, in the case of P‐CNFs current collectors, the morphology of deposited Li differed markedly (Figure 4c–i): no distinct Li dendrites were observed on the surface, instead Li filled the pores and covered the P‐CNFs current collectors. As the deposition capacity increased, the nanofiber diameter increased from 340 ± 69 to 390 ± 51, 430 ± 53, and 470 ± 59 nm at capacities of 1, 3, and 5 mAh cm^−2^ (Figure S27, Supporting Information), respectively. Cross‐sectional SEM images of the Li plated P‐CNFs nanofibers showed a thickness of ≈80 µm at deposition capacities of 1, 3, and 5 mAh cm^−2^ (Figure S28b–d, Supporting Information), thinner than the pristine P‐CNFs current collectors (120 µm, Figure S28a, Supporting Information) due to the coin cell's stacking pressure. This consistent thickness indicated homogeneous Li distribution within the P‐CNFs. SEM images with high magnification (Figure S28e–g, Supporting Information) of the nanofiber cross‐section at different orientations demonstrated similar morphology, further confirming uniform Li deposition. To examine the Li deposition morphology within the nanofibers, we captured the focused ion beam (FIB)/SEM image of the P‐CNFs current collectors after depositing 5 mAh cm^−2^ of Li, as shown in Figure 4f. It is found that the P‐CNFs are markedly different from the pristine state (Figure S29, Supporting Information) that the pores in the P‐CNFs are filled by deposited Li, suggesting the meticulously designed P‐CNFs with interconnected macropores can serve as stable Li‐ion flow rails to induce Li nucleation and deposition until they are completely filled with Li metal.^[^ ^78^ ^]^ With higher deposition capacity, Li started to fill the gaps between nanofibers and covered the surface of the nanofiber (Figure 4g–i). At 15 mAh cm^−2^, the plated nanofiber thickness was around 80 µm (Figure S30a, Supporting Information), similar to the calculated value of 72.6 µm, indicating high volume utilization due to the interconnected macropores and high porosity of the nanofibers. At 20 mAh cm^−2^ (Figure 4i), a homogeneous and flat Li layer was formed on the P‐CNFs current collector's surface with a thickness of ≈120 µm (Figure S30b, Supporting Information). Even at the maximum deposition capacity of 49 mAh cm^−2^, the plated nanofiber maintained a smooth and compact morphology (Figure 4j–l).

**Figure 5:**
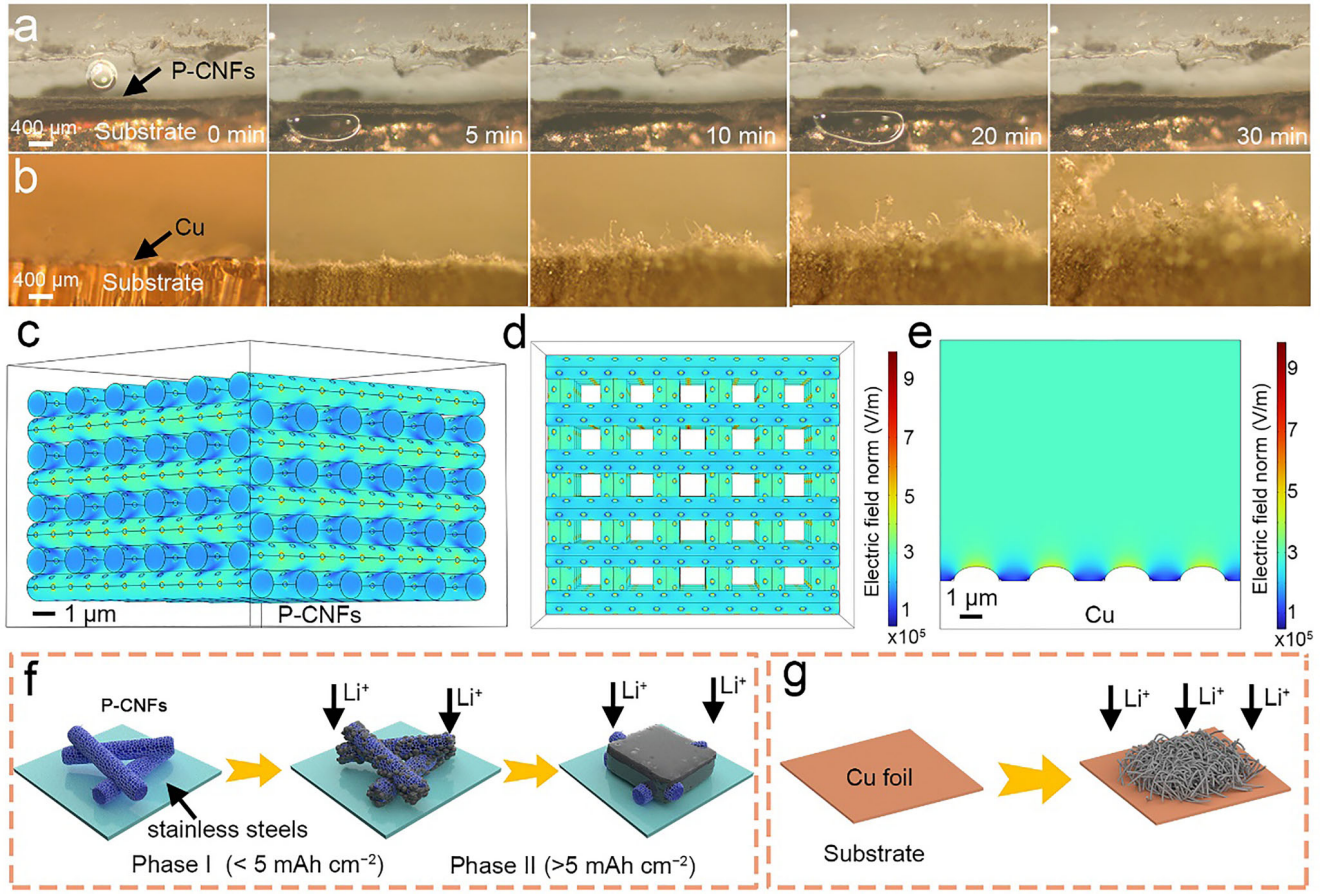
In situ optical photograph of electrodeposition of Li on a) P‐CNFs and b) Cu current collectors at a current density of 5 mA cm^−2^. Simulated electrical field distribution of P‐CNFs at c) cross‐section view and d) top‐view, and e) Cu foil. Schematic illustrations of the Li electrodeposition behavior on f) P‐CNFs and g) Cu current collectors.

The morphology of Li deposition on different current collectors was further observed by in situ optical microscope.^[^ ^79^ ^]^ **Figure** **5**a illustrates the Li deposition behavior on the P‐CNFs current collectors under a current density of 5 mA cm^−2^ at various time intervals (Figure S31, Supporting Information). Throughout the process, the P‐CNFs current collectors consistently achieve uniform Li plating. Even after reaching a deposition capacity of 2.5 mAh cm^−2^, no Li dendrites are observed. In contrast, mossy‐like Li dendrites appear on the surface of Cu foil only after 5 min (Figure 5b), and they quickly become conspicuous in upstanding rod shapes. This observation confirms that meticulously designed lithiophilic P‐CNFs current collectors with interconnected macropores effectively accommodate Li during plating, thereby enabling dendrite‐free Li deposition. To gain deeper insight into the distinct Li deposition behaviors in P‐CNFs and Cu, electrical field distribution during Li deposition was simulated using COMSOL Multiphysics 6.1. As illustrated in Figure 5c,d, the cross‐sectional and top‐view simulations of P‐CNFs reveal a homogeneous electrical field distribution, attributed to the porous structure enriched with lithiophilic sites. This feature has the potential to mitigate the concentration gradient of Li ions, thus alleviating the space charge layer shielding effect, thereby suppressing Li dendrite growth.^[^ ^80^ ^]^ In contrast, a localized region of high current density (indicated by the yellow areas) is observed on the protuberances of the rough Cu surface (Figure 5e). This uneven distribution promotes preferential Li deposition at these protrusions, subsequently triggering Li dendrite formation.

The morphology evolution of Li at different capacities verifies that the Li plating process on P‐CNFs current collectors can be divided into two phases: Phase I (Figure 5f), wherein Li metal starts to nucleate and grow inside the macropores of P‐CNFs current collectors. The pore channels and surface cavities act as preferential nucleation sites for Li plating because the heteroatom P doping changes the Li wettability of the carbon host from non‐wetting to superwetting. The plated Li finally fills up the pores and fully covers the carbon nanofibers without causing a volume change (<5 mAh cm^−2^); Phase II, as Li plating continues, Li deposition occurs uniformly along the surface of the P‐CNFs by the homogeneous electric field of 3D P‐CNFs host, forming a smooth and stable Li layer integrated with the SEI. The deposited Li grows beyond the filled voids, thickening the film and resulting in a volume change (>5 mAh cm^−2^). The reversible Li nucleation within the pores and cavities, coupled with the smooth Li plating in P‐CNFs, ensures dense and dendrite‐free Li deposition during cycling. However, due to the electron beam sensitivity of lithium, we were unable to obtain high‐resolution SEM images of the Li plating morphologies.^[^ ^81^ ^]^ In comparison, a bare Cu current collector tends to cause inhomogeneous Li nucleation and uncontrollable formation of Li dendrites, which finally cause early cell failure (Figure 5g).

**Figure 6:**
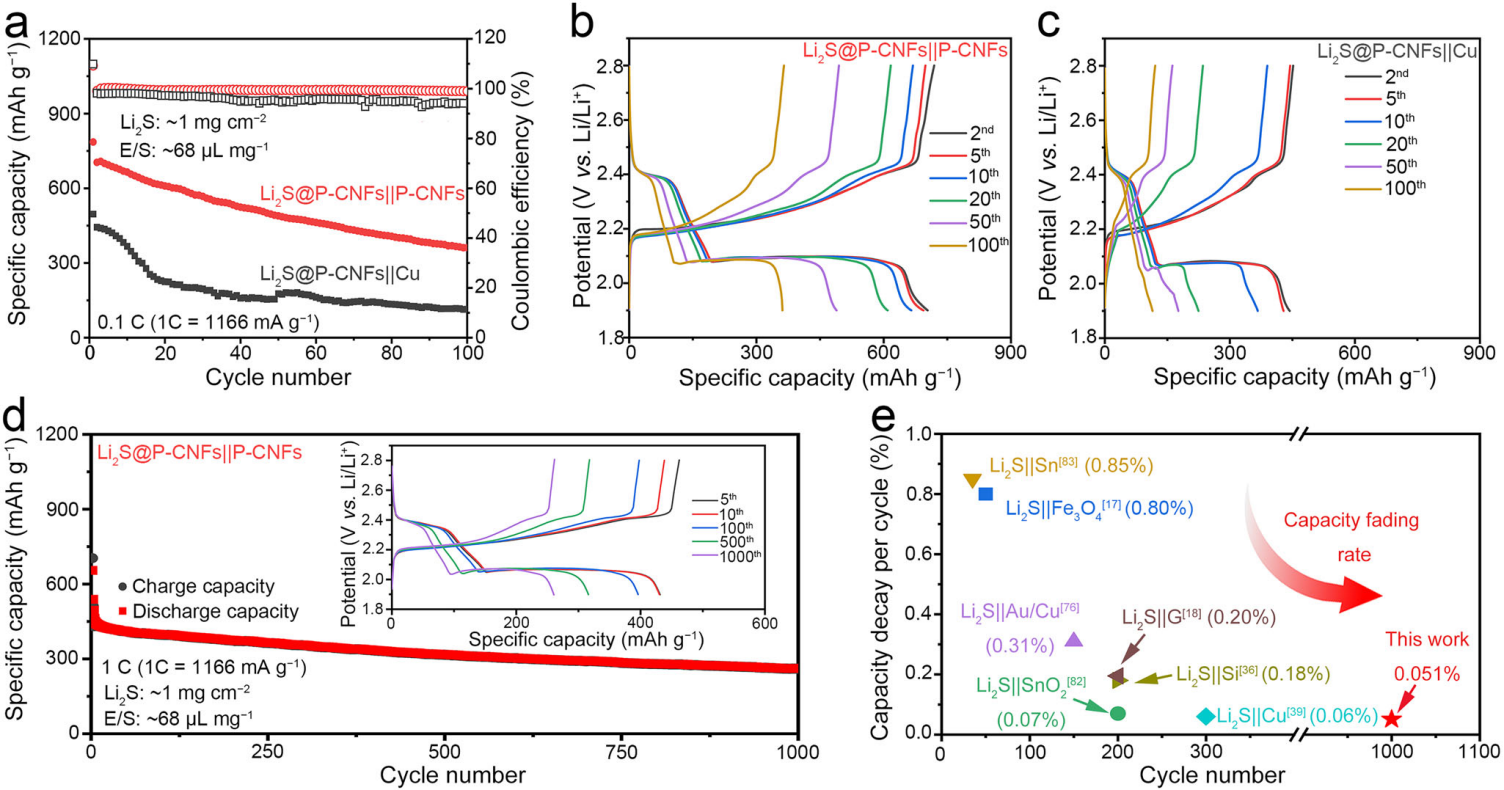
a) Specific discharge capacity of the Li_2_S@P‐CNFs||P‐CNFs and Li_2_S@P‐CNFs||Cu full cells at 0.1 C. GCD curves of the b) Li_2_S@P‐CNFs||P‐CNFs and c) Li_2_S@P‐CNFs||Cu full cells at different cycles. d) The specific capacity of the Li_2_S@P‐CNFs||P‐CNFs full cell at 1.0 C for 1000 cycles. e) Comparison of the capacity decay rate of different Li‐free Li–S batteries based on Li_2_S (Li_2_S||SnO_2_,^[^ ^82^ ^]^ Li_2_S||Fe_3_O_4_,^[^ ^17^ ^]^ Li_2_S||Au/Cu,^[^ ^76^ ^]^ Li_2_S||Sn,^[^ ^83^ ^]^ Li_2_S||Cu,^[^ ^39^ ^]^ Li_2_S||G,^[^ ^18^ ^]^ Li_2_S||Si^[^ ^36^ ^]^).

To further explore the potential application of the P‐CNFs in anode‐free Li–S batteries, the cycling performance of the Li_2_S@P‐CNFs||P‐CNFs and Li_2_S@P‐CNFs||Cu full cells were measured. Before making the full cell, the P‐CNFs and Cu current collectors first undergo three Li plating/stripping processes to improve the lithiophilicity of the current collectors and slow down the loss of initial Li deposition. This step, denoted as the lithiophilicity enhancement process, is illustrated in Figure S32 (Supporting Information). This process was terminated upon charging to 2.0 V versus Li/Li⁺, ensuring that all the lithium in the P‐CNFs or Cu was stripped. After that, the coin cells were disassembled and the pre‐lithiated P‐CNFs and Cu current collectors were taken out and coupled with Li_2_S@P‐CNFs to make the Li_2_S@P‐CNFs||P‐CNFs and Li_2_S@P‐CNFs||Cu full cells, respectively. The Li_2_S@P‐CNFs||P‐CNFs and Li_2_S@P‐CNFs||Cu full cells were first charged to 3.5 V at 0.05 C to activate Li_2_S cathode, similar to that of Li_2_S@P‐CNFs||Li half cells and then cycled at 0.1 C. As shown in **Figure** **6**a, the Li_2_S@P‐CNFs||P‐CNFs full cell delivered an initial discharge capacity of 785.7 mAh g^−1^ with a high initial Coulombic efficiency of 96.3% and this capacity dropped to 703.9 mAh g^−1^ at the second cycle due to the growth of new SEI and partially unstripped Li. To confirm SEI formation and formation of partially unstripped Li on our P‐CNFs electrode, we conducted SEM imaging under different electrochemical conditions: pristine P‐CNFs, P‐CNFs after the pre‐charge, and P‐CNFs after the first discharge of the Li_2_S@P‐CNFs||P‐CNFs full cell, as shown in Figure S33 (Supporting Information). Compared to the porous and rough morphology of the pristine P‐CNFs (Figure S33a, Supporting Information), the surface after pre‐charge (Figure S33b, Supporting Information) appears smooth and dense, suggesting that Li is deposited within the pores and uniformly covers the surface. After discharging the cell to 1.9 V, the morphology of the P‐CNFs (Figure S33c, Supporting Information) remains distinct from the pristine sample, which is attributed to the SEI formation on the electrode surface. Moreover, isolated Li was also observed within the complex porous architecture after the first discharge (Figure S33d, Supporting Information). These unstripped Li deposits, which become electronically disconnected from the current collector, lead to the formation of so‐called “dead Li.” The SEI layer and partially unstripped Li contribute to the rapid capacity fading observed during the initial cycles. In the following cycles, the discharge capacity of the Li_2_S@P‐CNFs||P‐CNFs full cell maintained at 361.4 mAh g^−1^ after 100 cycles at 0.1 C. As a comparison, the Li_2_S@P‐CNFs||Cu full cells exhibited an initial specific discharge capacity of 496.3 mAh g^−1^ and maintained at only 114.4 mAh g^−1^ for 100 cycles under the same conditions.

The enhanced specific discharge capacity of Li_2_S@P‐CNFs||P‐CNFs full cell is mainly due to the different Li deposition morphology on the anode side. In the Cu current collectors, dendritic or mossy Li plating will cause the continuous growth of SEI and the formation of dead Li, leading to low Coulombic efficiency and fast capacity decay.^[^ ^84^ ^]^ In the P‐CNFs current collectors, lithiophilicity and dendrite‐free Li deposition allow for better Li plating/stripping on P‐CNFs current collectors, resulting in higher Coulombic efficiency and cycling stability of the full cell. The GCD curves of the Li_2_S@P‐CNFs||P‐CNFs and Li_2_S@P‐CNFs||Cu full cells at different cycles are presented in Figure 6b,c, respectively, which show two pairs of plateaus in charge/discharge curves, similar to that of the half cells. The Li_2_S@P‐CNFs||P‐CNFs full cell delivered a stable discharge plateau and capacity throughout the cycle. On the contrary, the second discharge plateau of the Li_2_S@P‐CNFs||Cu full cell (roughly at 2.1 V, accounting for 75% of theoretical capacity and encompassing the most kinetically sluggish solid‐phase conversion^[^ ^67^, ^68^, ^69^ ^]^) was stable only at the initial three cycles and then rapidly decayed to a very low capacity, due to the irreversible Li loss. To confirm this, the Li_2_S@P‐CNFs||Cu full cell after cycling was disassembled and the Cu current collectors were extracted for SEM measurement. As shown in Figure S34 (Supporting Information), dead Li is observable on the Cu current collectors after cycling, suggesting that the dead Li caused by the mossy Li deposition on Cu current collectors and the sluggish solid‐phase conversion reactions are the primary reasons for the fast capacity decay.

The electrochemical performance of Li_2_S@P‐CNFs||P‐CNFs full cell at a high Li_2_S loading of 3 mg cm^−2^ and electrolyte to sulfur (E/S) ratio of 22.6 µL mg^−1^ has been measured. As shown in Figure S35 (Supporting Information), the Li_2_S@P‐CNFs||P‐CNFs full cell delivered a discharge capacity of 527.3, 453.1, and 325.4 mAh g^−1^ at current densities of 0.1, 0.2, and 0.5 C, respectively. A reversible specific discharge capacity of 359.5 mAh g^−1^ was achieved when the current density was shifted back to 0.2 C, demonstrating its good rate capability. After repeated cycling at varying current densities, the discharge capacity of the Li_2_S@P‐CNFs||P‐CNFs full cell can be maintained at 265.4 mAh g^−1^ after another 50 cycles at 0.2C, demonstrating its outstanding capacity and stability. The cycling performance of the Li_2_S@P‐CNFs||P‐CNFs full cell was further demonstrated in long‐term cycling at 1.0 C, as illustrated in Figure 6d. The Li_2_S@P‐CNFs||P‐CNFs full cell displayed an initial specific discharge capacity of 654.6 mAh g^−1^ and dropped to 538.5 mAh g^−1^ at the second cycle. Even after 1000 cycles, the specific discharge capacity can still be maintained at 261.5 mAh g^−1^, corresponding to a low capacity decay of 0.051% cycle^−1^ (calculated from the second cycle). The improved cycling stability of the Li_2_S@P‐CNFs||P‐CNFs full cell has been compared to the previously reported Li_2_S‐based Li‐free batteries with various configurations (Figure 6e and Table S2, Supporting Information). The Li_2_S@P‐CNFs||P‐CNFs full cell exhibits the lowest capacity decay rate compared to previously reported works, primarily due to the following reasons: (1) The nanosized Li_2_S‐loaded porous P‐CNFs efficiently lower the activation barrier of Li_2_S, improving active material utilization. (2) The P doping facilitates unimpeded electron/ion transport at the polar carbon matrix interface, thus enhancing the Li_2_S conversion reaction kinetics and mitigating the shuttling effect of LiPSs during cycling. (3) The lithiophilic P‐CNFs current collectors with interconnected macropores promoted uniform Li plating behavior, resulting in smooth, compact deposition morphology and improved Li plating/stripping efficiency. The energy density of the assembled full cell based on the total mass of the electrodes is calculated to be 469.0 Wh kg^−1^ (details can be found in Equation (S4), Supporting Information). Although the Li_2_S loading and E/S ratio used in our study are not yet at the level of practical Li–S pouch cells, the obtained energy cycling stability (up to 1000 cycles) and energy density (469.0 Wh kg^−1^ based on electrode mass) are highly competitive. Further optimization toward high‐loading cathodes and lean‐electrolyte configurations will be essential for practical application.

## 3 Conclusion

In summary, we have proposed a novel anode‐free Li–S battery configuration featuring nanosized Li_2_S embedded in P‐doped porous carbon nanofibers (Li_2_S@P‐CNFs) as a freestanding cathode, with P‐CNFs serving as the Li host in the anode side. In the cathode, the in situ formation of nanosized Li_2_S within the polar P‐CNFs enhances the kinetics of the Li_2_S redox reduction reaction and mitigates the polysulfide shuttle effect. On the anode side, unlike the conventional anode‐free Li–S battery configuration, which employs a heavy Cu metal current collector, P‐CNFs can work efficiently as the host for Li. SEM images, in situ optical microscopy, and finite element simulations collectively demonstrate that the lithiophilic P‐CNFs effectively homogenize the electric field distribution and accommodate substantial amounts of Li during the Li plating process, leading to dendrite‐free Li deposition and significantly enhance the efficiency of Li plating/stripping. Even at a high deposition capacity of 49 mAh cm^−2^, the plated nanofiber maintained a smooth and compact morphology, free from Li dendrites, highlighting the well‐regulated Li deposition facilitated by the lithiophilic P‐CNFs current collectors with interconnected macropores. Benefitting from the synergistic advantages, the coupled anode‐free Li–S full cell (Li_2_S@P‐CNFs || P‐CNFs) exhibits ultra‐long cycling life (1000 cycles at 1 C) with a low‐capacity decay of 0.051% cycle^−1^ (calculated from the second cycle). Future work needs to increase the active material loading through post‐infiltration or melt‐diffusion techniques, which are expected to substantially improve the energy density without compromising the structural advantages of the bifunctional host. Furthermore, the exceptional cycling stability demonstrated in our system, particularly under the demanding anode‐free configuration, highlights the rationality and robustness of our design strategy. This work could offer an effective route to solve the obstacles for both S cathode and Li anode to achieve stable anode‐free Li–S batteries with long cycle life.

## Conflict of Interest

The authors declare no conflict of interest.

## Supporting information



Supporting Information

Supplemental Video 1

## Data Availability

The data that support the findings of this study are available from the corresponding author upon reasonable request.
