# Supplementary material for: Nanosized Li2S‐Loaded Polar Porous Carbon Nanofibers as Self‐Supporting Electrodes in Anode‐Free Lithium–Sulfur Batteries
Source: Adv Sci (Weinh). 2025 Dec 8;13(5):e16575. doi: 10.1002/advs.202516575 (PMC12850093; doi:10.1002/advs.202516575)
Supplement: Supplementary file 1 — Supporting Information [file ADVS-13-e16575-s002.docx]

Support Information for

**Nano-sized Li_2_S-loaded Polar Porous Carbon Nanofibers as Self-supporting Electrodes in** **Anode-free Lithium−Sulfur Batteries**

*Ping Feng^a,b^, Qingping Wu^a,c^, Yaolin Xu^a^, Liqiang Lu^a^, Tianle Zheng^d^, Tonghui Xu^e^, Wen Xu^f^, Daniel Höche^f^, Zdravko Kochovski^a^, Yan Lu^a,b,g*^*

P. Feng, Q. Wu, Y. Xu, L. Lu, Z. Kochovski, Y. Lu

^a^Institute of Electrochemical Energy Storage, Helmholtz-Zentrum Berlin für Materialien und Energie, Berlin,14109, Germany.

P. Feng, Y. Lu

^b^Institute for Technical Chemistry and Environmental Chemistry, Friedrich-Schiller-Universität Jena, Jena, 07743, Germany.

Q.Wu

^c^Chongqing Institute of Green and Intelligent Technology, Chinese Academy of Sciences, Chongqing, 400714, China.

T. Zheng

^d^School of Natural Sciences, Technical University of Munich (TUM), James-Franck-Str. 1, Garching, 85748, Germany.

T. Xu

^e^Department of Chemistry, College of Sciences, Shanghai University, Shanghai, 200444, P. R. China.

W. Xu, D. Höche

^f^Institute of Surface Science, Helmholtz-Zentrum Hereon, Max-Planck-Str. 1, Geesthacht, 21502, Germany.

Y. Lu

^g^Helmholtz Institute for Polymers in Energy Applications Jena (HIPOLE Jena), Jena, 07743, Germany.

**Materials:** Sodium styrene sulfonate (NaSS, 97.0%), polyvinyl pyrrolidone (PVP, average Mw = 1,300,000), lithium sulfate monohydrate (Li_2_SO_4_·H_2_O, 99.0%), ethanol absolute (CH_3_CH_2_OH, 99.8%), phosphoric acid solution (H_3_PO_4_, 85% in water), bis(trifluoromethane)sulfonimide lithium salt (LiTFSI, 99.95 %), 1,3-dioxolane (DOL, 99.0 %), 1,2-dimethoxyethane (DME, 99.0 %), lithium nitrate (LiNO_3_, 99.99%), sublimed sulfur powder, lithium sulfide (Li_2_S, 99.98%), polyvinylidene fluoride (PVDF) were purchased from Sigma-Aldrich. All chemicals were used without any further purification.

**Synthesis of the Spherical Polyelectrolyte Brush (SPB):** SPB with sodium polystyrene sulfonate brushes was synthesized in a two-step polymerization process according to our previous work.^[1]^ Polystyrene (PS) core particles were first prepared with a thin layer of 2-[p-(2-Hydroxy-2-methylpropiophenone)]ethylene glycol methacrylate (HMEM) photo-initiator on the surface by emulsion polymerization. Then the PS-HMEM particles were cleaned through ultrafiltration until the conductivity of the serum reached 20~30 μS cm^−1^. NaSS was chosen as the monomer, and the polyelectrolyte chains were grafted onto the PS cores by photo-emulsion polymerization. In a typical run, 11.58 g sodium styrene sulfonate was slowly added to 780 ml PS-HMEM solution (solid content: 2.5 wt %), followed by the removal of O_2_ through purging with N_2_. UV irradiation was then applied for photo-emulsion polymerization using a UV reactor (Heraeus TQ150 Z3, range of wavelength 200–600 nm) at room temperature for 60 min. After that, the SPB particles were cleaned by ultrafiltration till the conductivity of the filtrate reached between 3 μS cm^−1^ and 5 μS cm^−1^.

**Synthesis of the nano-sized Li_2_S embedded in P-doped porous carbon nanofibers (Li_2_S@P-CNFs):** The Li_2_S@P-CNFs were prepared by the electrospinning method (Spinbox System, Bioinicia S.L.). The details are shown below: 0.8 g PVP and 0.6 g Li_2_SO_4_·H_2_O were directly dissolved in a mixed solution of 4 mL CH_3_CH_2_OH, and 6 ml SPB aqueous colloidal dispersion (solid content: 5.6 wt %) and stirred mildly for 4 hours. 100 μL H_3_PO_4_ solution (85 wt% in water) was added as the phosphorus source. We explored the effect of various phosphorus doping levels by adding different amounts of H_3_PO_4_ solution (50, 100, and 150 μL) into 6 mL of aqueous SPB colloidal dispersion (solid content: 5.6 wt%). However, when 150 μL of H_3_PO_4_ was added to the 6 mL SPB dispersion in the presence of 0.6 g of Li_2_SO_4_·H_2_O, we observed significant aggregation of SPB particles. This is likely due to the strong electrostatic interactions among SPB, H_3_PO_4_, and Li_2_SO_4_, which disrupted the colloidal stability. We also measure the electrochemical performance of the cathode synthesized with 50 μL H_3_PO_4_ (denoted as Li_2_S@P-CNFs-50). As shown in **Figure S23**, the Li_2_S@P-CNFs-50 electrode exhibited an initial specific discharge capacity of 531.6 mAh g^−1^ at 0.2 C, which decreased to 377.2 mAh g^−1^ after 100 cycles, corresponding to a capacity retention of 70.9 %. Both the specific discharge capacity and the retention rate are lower than those of the Li_2_S@P-CNFs electrode. Based on these observations, we selected 100 μL of H_3_PO_4_ as the optimized doping level to achieve a balance between improved electrochemical performance and colloidal stability. This solution was loaded into a syringe with a needle and connected to a pump to control the flow rate. The distance between the needle and the collector was 15.0 cm and the electrospun process was conducted by a constant flow of 1.0 mL h^−1^ under the voltage of 12.0 kV. Afterwards, the obtained Li_2_SO_4_@SPB@PVP nanofibers were peeled off and dried in the oven at 80 °C overnight to evaporate the residual solvent. Subsequently, the collected polymer fibers were peroxided in air at 150 °C for 2 h. Finally, the polymer nanofibers were calcined at 900 °C for 2.0 h under an Ar atmosphere at a ramping rate of 5.0 °C/min to get the Li_2_S@P-CNFs. Two control samples were prepared using the same method as Li_2_S@P-CNFs, but one without Li_2_SO_4_ to obtain P-doped CNFs (P-CNFs) and the other without PA to obtain Li_2_S nanoparticles embedded in pure CNFs (Li_2_S@CNFs).

**Electrochemical measurements:** For the half-cell measurement**,** CR2032 coin cells were assembled with the Li_2_S@P-CNFs (or Li_2_S@CNFs) as the cathode, the Li foil as the anode, and a piece of Celgard membrane as the separator in an Ar-filled glove box (UNIlab plus, M. BRAUN) with H_2_O content < 0.5 ppm and O_2_ content < 0.5 ppm. 1.0 M LiTFSI in a 1:1 volume ratio of DOL/DME with 2.0 wt. % of LiNO_3_ was used as the electrolyte. The cathode side was supplemented with 30 μL electrolyte, and the anode side was supplemented with 15.0 μL electrolyte. The Li_2_S loading in the cathodes is ~1.0 mg cm^−2^, and the electrolyte-to-sulfur (E/S) rate is ~68 μL mg^−1^. Before the electrochemical testing, all the cells were aged at room temperature under open-circuit potential for 12.0 h to allow the electrolyte to wet the electrode. In this work, the current density of 1.0 C equals 1,166.0 mA g^−1^. The specific capacity is calculated based on the mass of Li_2_S. The galvanostatic charge and discharge were conducted on a Neware battery testing system at room temperature. The half cells were first charged to 3.5 V to activate the Li_2_S nanoparticles and then cycled at the specific current densities from 1.9 V to 2.8 V. The CV curves of the assembled coin cells were measured with a Biologic VMP3 electrochemical workstation. For the Coulombic efficiency measurement, P-CNFs or Cu were used as the anode and Li metal as the cathode, respectively. Before cycling, the half cells were preconditioned with 3 cycles at 0.1 mA cm⁻^2^ from 0.1 V to 2.0 V to establish a relatively stable SEI on the current collectors (**Figure S25**). For the full cell measurement, the P-CNFs and Cu current collectors were first paired with Li metal anode to undergo three Li plating/stripping processes at 1 mA cm^−2^ to improve the lithophilicity of the current collectors, as shown in **Figure S32**. After that, the coin cell was disassembled, and the P-CNFs and Cu current collectors were taken out to pair with Li_2_S@P-CNFs for full cell measurements. The cathode side and the anode side were supplemented with 30.0 μL fresh electrolyte, respectively. Before the electrochemical testing, all the cells were aged at room temperature under open circuit potential for 12.0 h to let the electrolyte wet the electrode. The full cells were first charged to 3.5 V to activate the Li_2_S nanoparticles and then cycled at the specific current densities from 1.9 V to 2.8 V. The specific capacity is calculated based on the mass of Li_2_S. The galvanostatic charge and discharge were conducted on a Neware battery testing system at room temperature.

**Adsorption tests of LiPSs:** A Li_2_S_6_ solution was prepared by dissolving the appropriate amounts of sulfur and Li_2_S powder in a DOL/DME solution (1:1 by volume). The solution was then stirred at 80 °C for 48 hours inside a glove box to ensure complete reaction. After that, the powder of P-CNFs and CNFs particles with the same mass (20.0 mg) were added to 2.0 mM Li_2_S_6_ solution (4.0 mL), respectively. After aging for 3.0 hours inside the glove box, the supernatant liquid was sealed in a quartz cylinder for the UV-vis absorption spectroscopy test.

**Kinetics of Li_2_S precipitation on the host materials:** The Li_2_S_8_ catholyte was prepared by the chemical reaction between sulfur and lithium sulfide (Li_2_S + 7S → Li_2_S_8_). In a typical process, 4.48 g sulfur and 0.92 g Li_2_S (99.98%) were dissolved in 20.0 mL DOL/DME solution (V_DOL_: V_DME_ = 1: 1) with 2.0 wt. % LiNO_3_ additives in a 50.0 mL bottle and kept stirring overnight in an Ar-filled glove box. Then this suspension was heated at 80 °C in a vacuum oven inside the glove box for one day to yield the Li_2_S_8_ catholyte (1.0 M) with red-brown color. For the Li_2_S precipitation test, the electrode was prepared by casting the slurry of P-CNFs (or CNFs), conductive carbon, and PVDF (7:2:1 in weight ratio) on carbon paper by the doctor blade technique. After drying at 50 °C under vacuum overnight, the electrode was cut into wafers with a diameter of 12.7 mm. The coin cell was assembled with the P-CNFs (or CNFs) as the cathode, Li as the anode, and a Celgard membrane serving as the separator. The loading of Li_2_S_8_ catholyte for the Li_2_S precipitation test is 1 mg cm^−2^. The cathode and the anode sides were supplemented with 15.0 μL of electrolytes, respectively. All the assembled coin cells were aged at room temperature for 12.0 h. After that, the cell was first discharged galvanostatically at 0.1 C to 2.12 V and then discharged potentiostatically at 2.05 V for Li_2_S nucleation and growth. The current *vs.* time curve was collected for kinetic analysis. For the symmetrical cell, two identical electrodes (P-CNFs or CNFs) were assembled into a CR2032 coin cell with a Celgard membrane serving as the separator. 1.0 M Li_2_S_8_ catholyte (5.0 μL) was loaded onto the host electrodes as the sulfur source. The cathode and the anode sides were supplemented with 15.0 μL of electrolytes, respectively. CV measurements of the symmetric cell were performed at a scan rate of 10.0 mV s^−1^ within the potential range from –1.0 to 1.0 V.

**Characterization:** The morphology of the obtained samples was investigated by a LEO 1530 field emission SEM and a JEOL-2100 TEM (JEOL GmbH, Germany) operated at 200 kV. FIB/SEM was conducted with Zeiss Crossbeam 340 at room temperature. SEM images were taken primarily at 3 kV. FIB cutting was performed with a gallium ion beam voltage of 30 kV. The current used for FIB cutting was 50 pA. For the [electron tomography](https://www.sciencedirect.com/topics/materials-science/electron-tomography), [nanofibers](https://www.sciencedirect.com/topics/chemical-engineering/nanoparticle) were dispersed in ethanol and then loaded on lacey copper TEM grids coated with carbon (200 mesh, Science Services). After drying, the tomographic images were recorded using a JEM-2100 TEM equipped with a 4k × 4k CMOS digital camera (TVIPS TemCam-F416). The operation voltage is 200 kV. With the Serial-EM acquisition software package, tilt series were acquired with a 2° angular increment and a tilt range of ±60° at magnification 30,000×, representing 3.8 Å per pixel size at the specimen level. With the IMOD software package, tilt series were arranged by patch tracking and rebuilt by weighted back-projection. Surface segmentations were performed manually using Amira (FEI Company, Netherlands). UCSF Chimera was used to generate 3D surface renderings and videos. The diameter analysis of carbon nanofibers was conducted using ImageJ.

X-ray diffraction (XRD) patterns were collected in Bragg-Brentano geometry on a Bruker D8 Advance diffractometer with Cu_Kα_ radiation using a zero-background holder and a step size of 0.03 °/step and a measuring time of 1 s/step. N_2_ adsorption-desorption isotherms were conducted by using Quantachrome Autosorb-1 systems at 77 K. Specific surface areas were calculated by using the Brunauer-Emmett-Teller (BET) method based on a multipoint analysis. The chemical states of the elements in the samples were characterized using X-ray photoelectron spectroscopy (XPS) with an ESCA-Lab-220i-XL X-ray Photoelectron Spectrometer (Thermo Fisher Scientific) with Al Kα sources (*hν* = 1,486.6 eV). Thermogravimetric analysis (TGA) is carried out in a Netzsch TG209 F1 analyzer under an Ar (or synthetic air) stream. The amount of the Li_2_S in Li_2_S@P-CNFs was calculated using the formula wt% (Li_2_S) = wt% (Li_2_SO_4_) × M(Li_2_S)/M(Li_2_SO_4_). Dynamic light scattering (DLS) was measured by using a Malvern Zetasizer Nano ZS. The scattered light was detected at an angle of 173°. Raman spectroscopy was conducted with a Renishaw InVia confocal Raman microscope with a 532 nm laser. The elemental composition of the samples was analyzed using inductively coupled plasma mass spectrometry (ICP-MS, Agilent 7900, Agilent Technologies, USA).

***In-situ* optical microscopic observations:** *In-situ* optical microscopic observations were carried out in the home-made optical cell with a quartz window for observation. The assembly of optical cells was performed at room temperature in an Argon-filled glovebox with H_2_O content < 0.5 ppm and O_2_ content < 0.5 ppm. The Li anode was placed into the cells. After injecting electrolyte, the cell was sealed in the Argon-filled glovebox. Optical microscope with a fitted charge coupled device (CCD) camera was applied to shoot the Li deposition process.

**Computational methods:** The density functional theory (DFT) method was employed by CASTEP with GGA/PBE functional, custom Grimme DFT-D parameters with ultrasoft pseudopotential in Material Studio software.^[2]^ All molecules (Li_2_S_y_, Graphite, P-doped Graphite) were under ultra-fine optimization. The k-points were set as Gamma (2 × 2 × 1) with a kinetic energy cutoff of 520 eV, and convergence tolerance criteria of 1.0 × 10^-5^ eV for energy, 0.01 EV/ Å^−1^ for maximum force,0.02 GPa for maximum stress, and 5.0 × 10^-4^ Å for maximum displacement were used. The adsorption energy was calculated by the following formula S1:

$E_{ads}= E_{total}-E_{slab}- E_{molecule}$ S1

where E_total_, E_slab_, and E_molecule_ were the free energy of total structure, different surfaces (Graphite or P-Graphite) and Li_2_S_y_, respectively.^[3]^ The free energy was calculated using the equation S2:

$G= E_{ads}+ZPE- TS$ S2

where G, E_ads_, ZPE, and TS are the free energy, total energy from DFT calculations, zero-point energy, and entropic contributions, respectively.

**Finite element simulations**: The finite element simulations were conducted on COMSOL Multiphysics 6.1. Simplified models were used to simulate the electric field for P-CNFs and Cu foil. In the case of P-CNFs, a 6 × 8 nanofiber array with a gap of 1000 nm along both X and Y directions was modeled as the P-CNFs electrode. The diameter of the fibers was set at 500 nm according to the SEM images. The electrical conductivity of the P-CNFs, Cu, and electrolyte was set to 10, 5.8 × 10^5^, and 1 S cm^−1^, respectively. The initial Li^+^ concentration was set to 1.2 mol L^−1^. The positive electrode is bound by zero potential, and the voltage difference between the positive and negative electrode is set to 2.8 V.


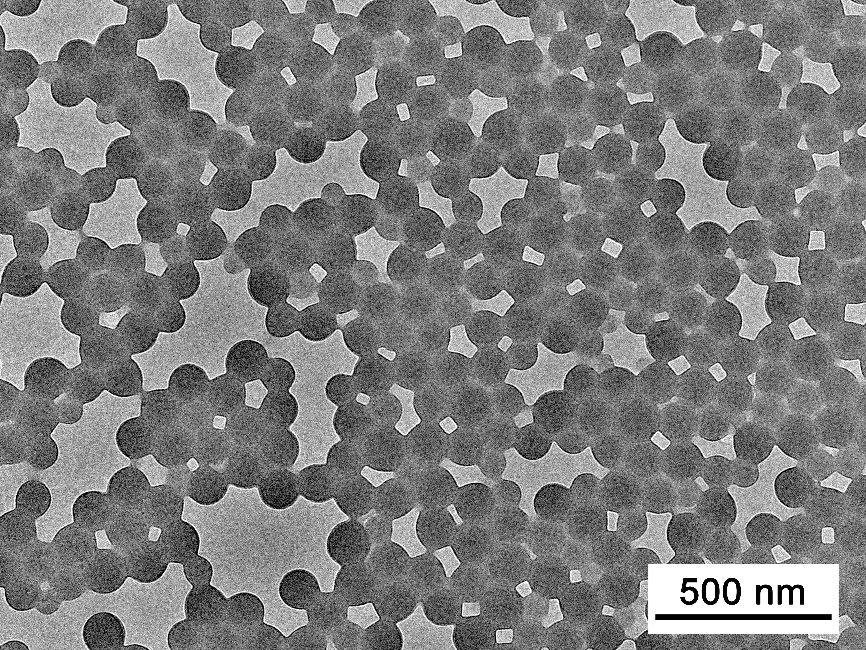


**Figure S1**. TEM image of the as-prepared SPB nanospheres. No brushes were detected on the surface of SPB due to the “dry effect” during TEM sample preparation.^[4]^


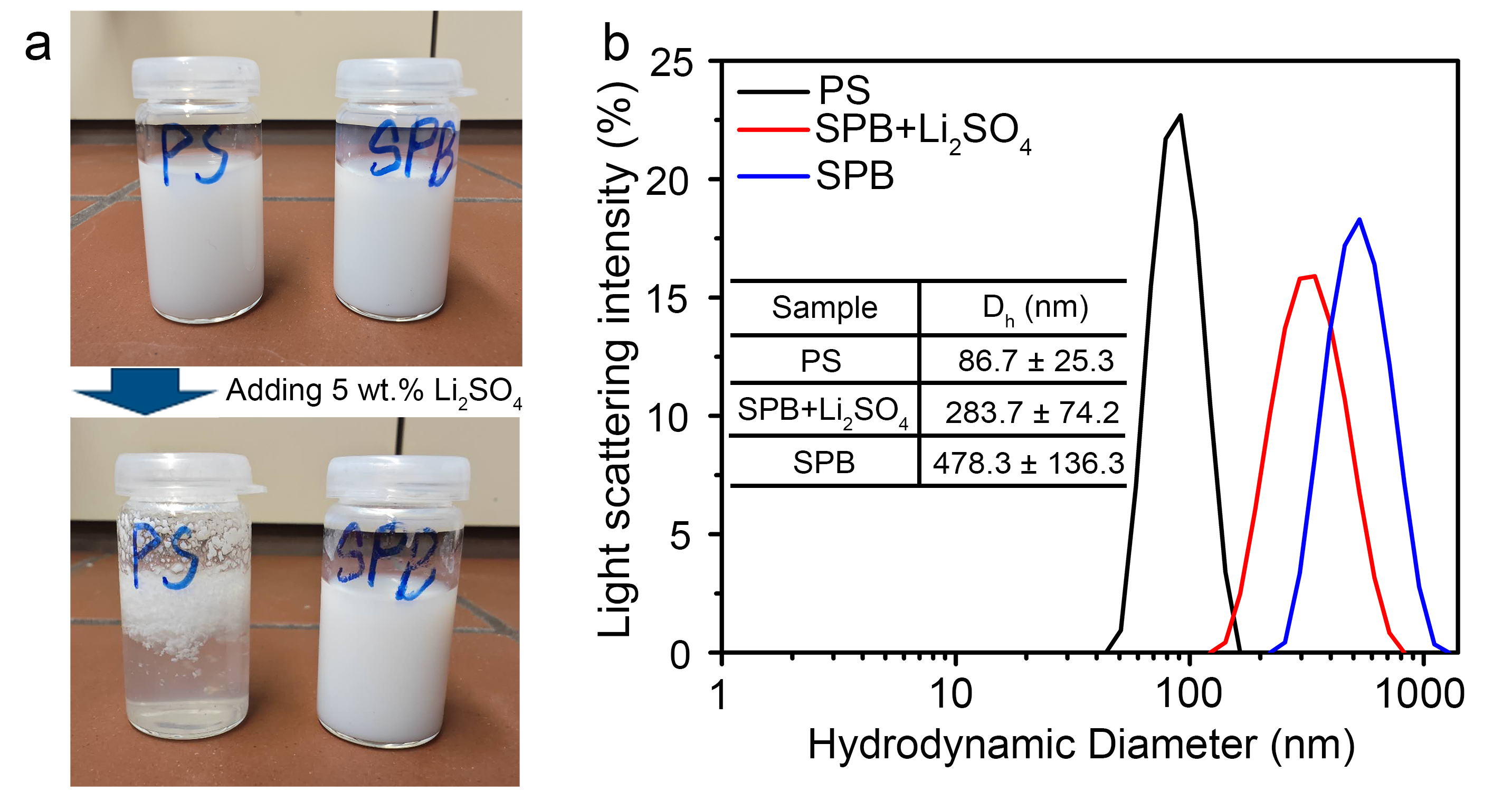


**Figure S2**. (a) Digital images of the aqueous PS dispersion and SPB dispersion before (top) and after (bottom) mixing with 5 wt.% of Li_2_SO_4_. (b) Dynamic light scattering of the aqueous PS dispersion, SPB dispersion, and SPB dispersion with 5 wt.% of Li_2_SO_4_.


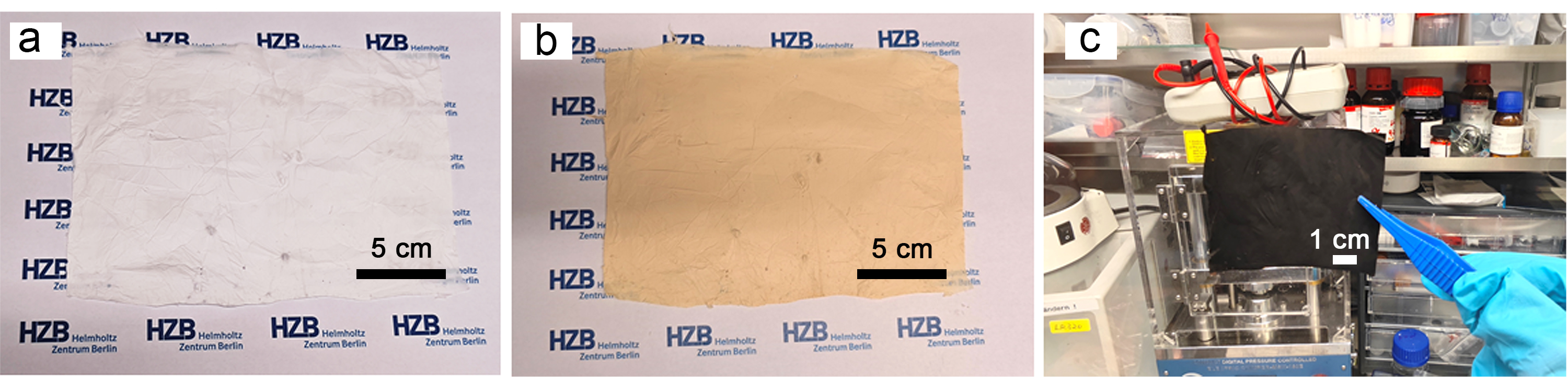


**Figure S3**. Digital images of the (a) as-prepared polymer nanofibers, (b) pre-oxidized Li_2_SO_4_@SPB@PVP nanofibers, and (c) Li_2_S@P-CNFs nanofibers.


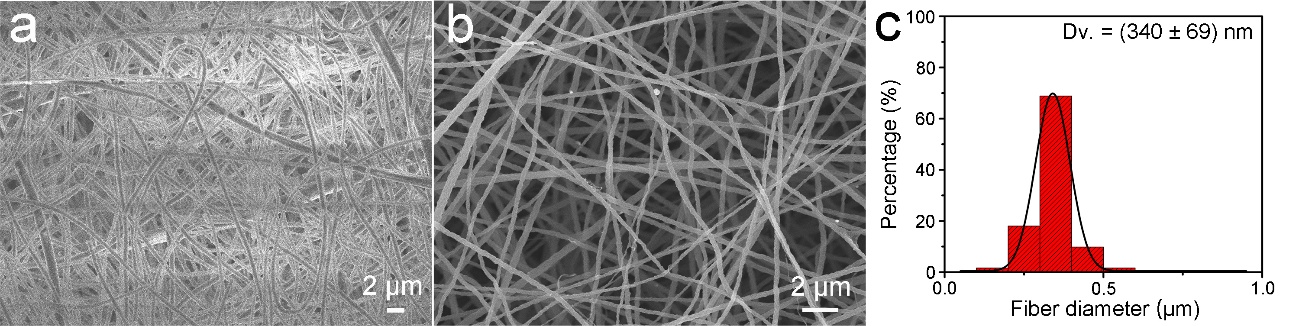


**Figure S4**. SEM images of the (a) pre-oxidation Li_2_SO_4_@SPB@PVP nanofibers, (b) Li_2_S@P-CNFs nanofibers at low magnification, and (c) diagram of statistical analysis of the average diameter of the Li_2_S@P-CNFs nanofibers from their SEM images.


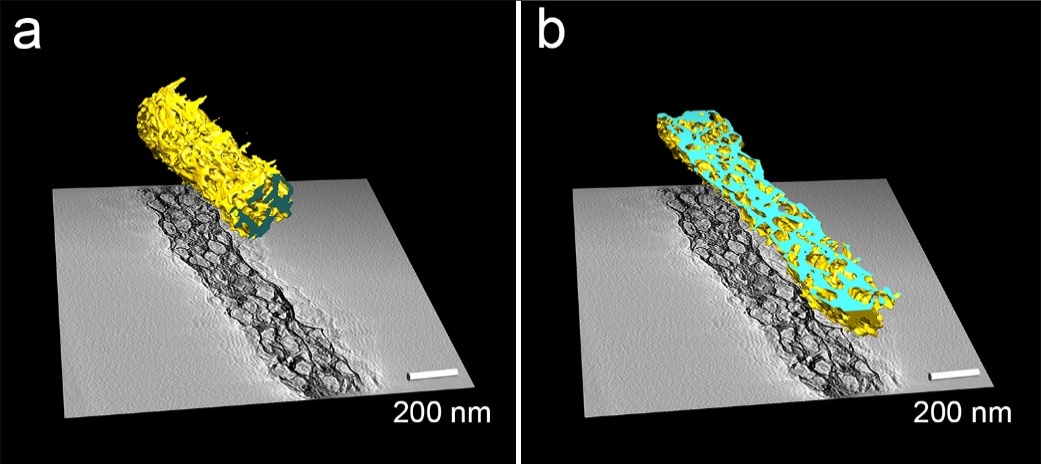


**Figure S5**. Three-dimensional volume renderings from TEM tomographic reconstruction of a single P-CNFs nanofiber, shown over a central XY slice from the reconstruction, with cross-sectional views in (a) the parallel direction and (b) the perpendicular direction.


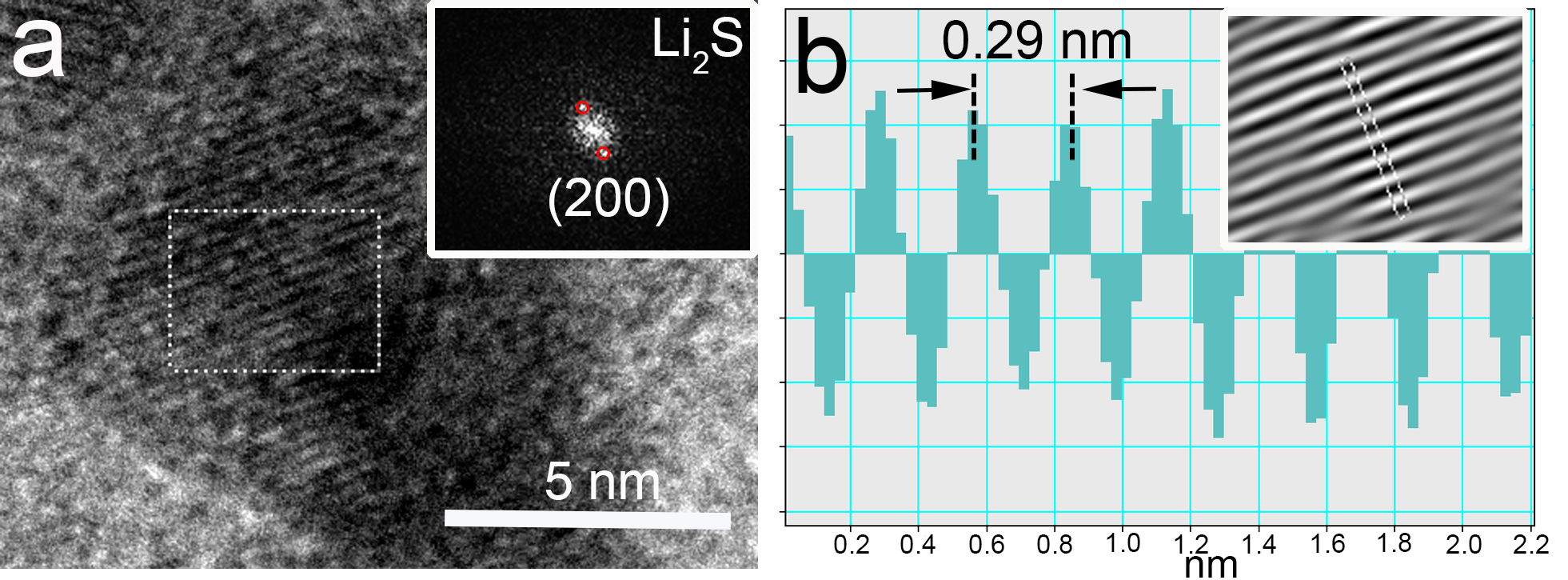


**Figure S6**. (a) High-resolution TEM image of the Li_2_S@P-CNFs. (b) Line profile for the selected line in its inverse FFT image.


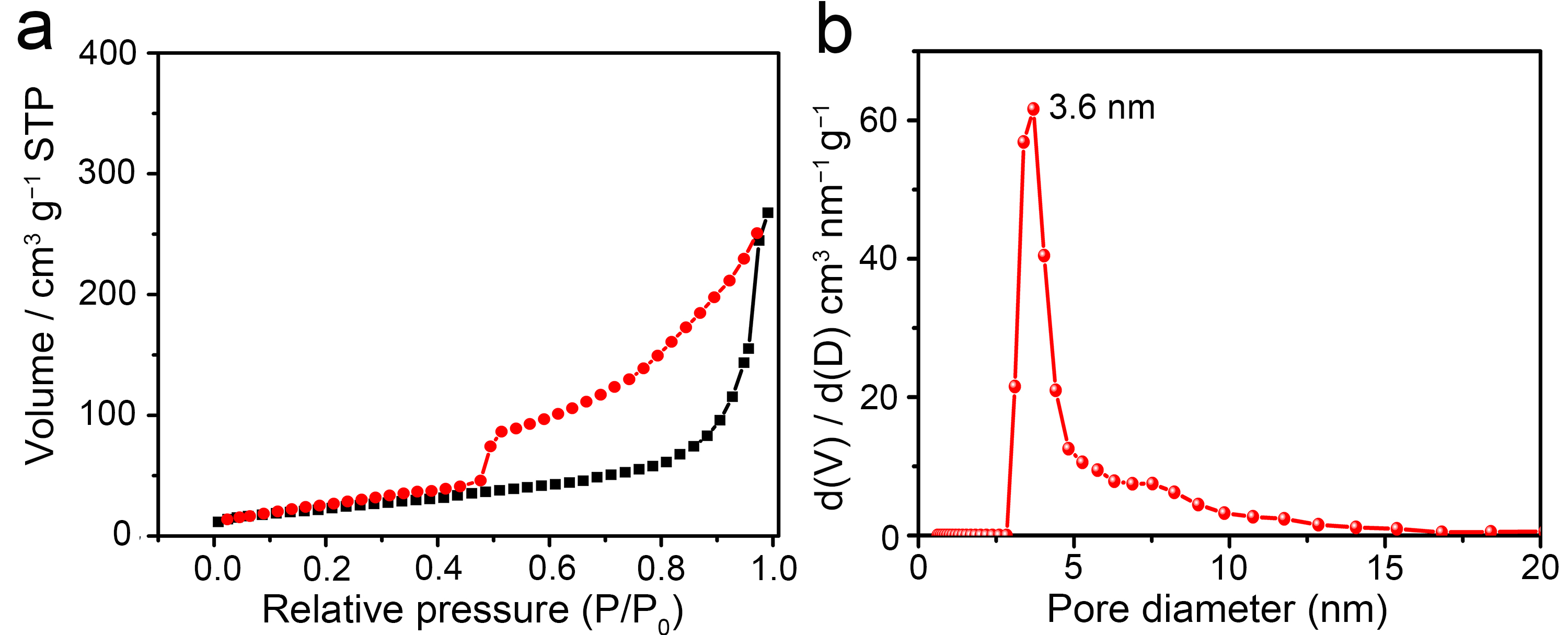


**Figure S7**. (a) Nitrogen adsorption−desorption isotherms and (b) the corresponding pore size distribution plot of the Li_2_S@P-CNFs nanofibers.


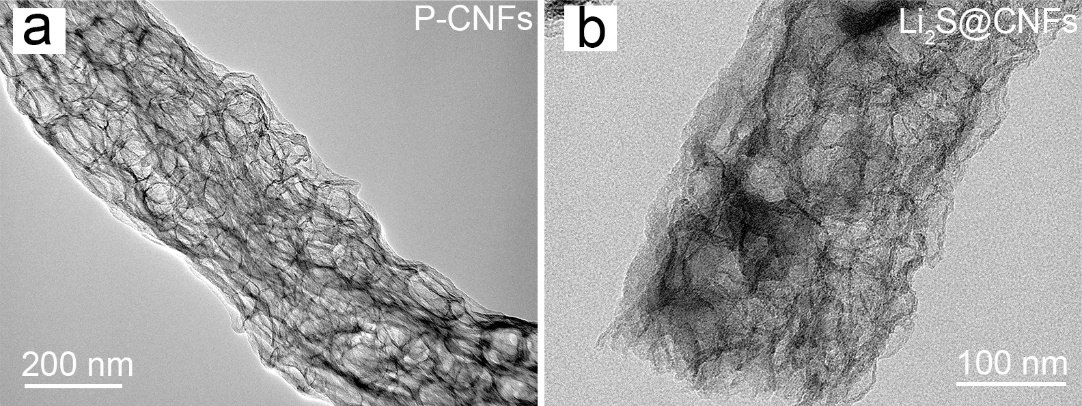


**Figure S8**. TEM images of the (a) P-CNFs and (b) Li_2_S@CNFs.


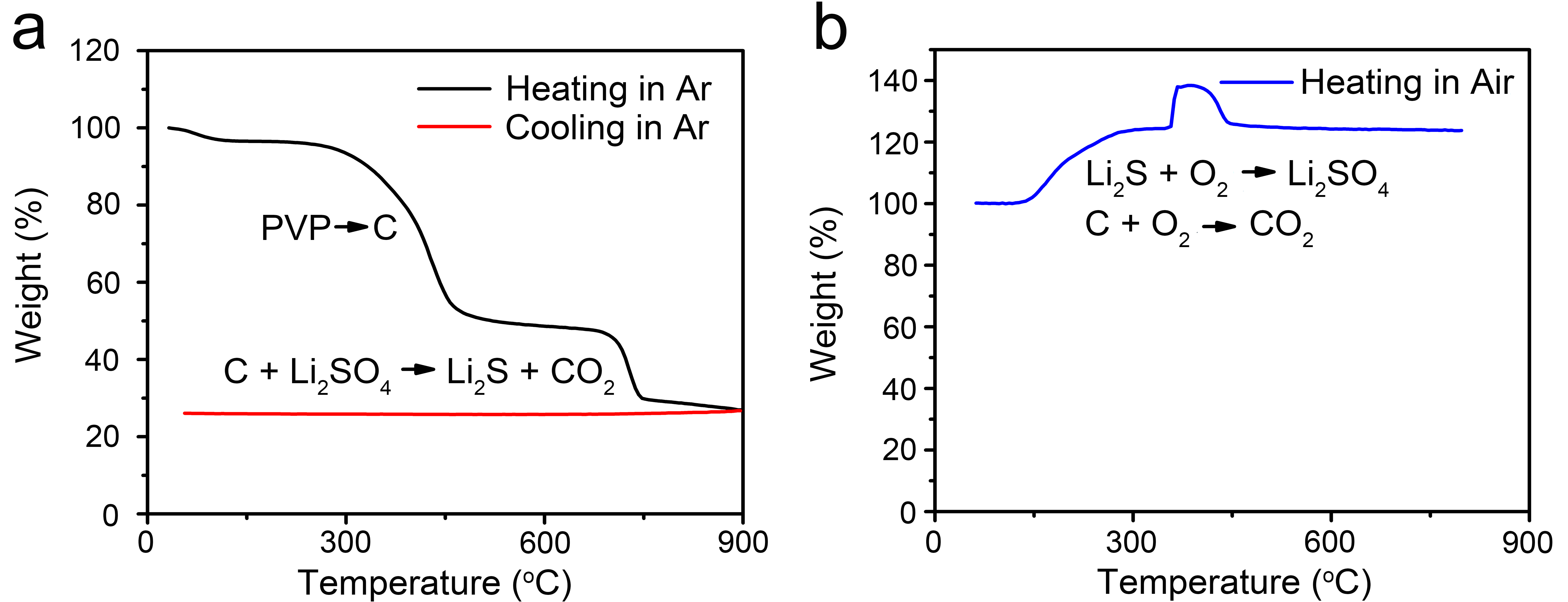


**Figure S9**. TGA curves of (a) the initial heating of the Li_2_SO_4_@SPB@PVP nanofibers from room temperature to 900 °C under Ar flow at a rate of 5 °C min^−1^ and then cooling to room temperature in Ar, and (b) the following heating of Li_2_S@P-CNFs to 800 °C under dry Air at a rate of 5 °C min^−1^.


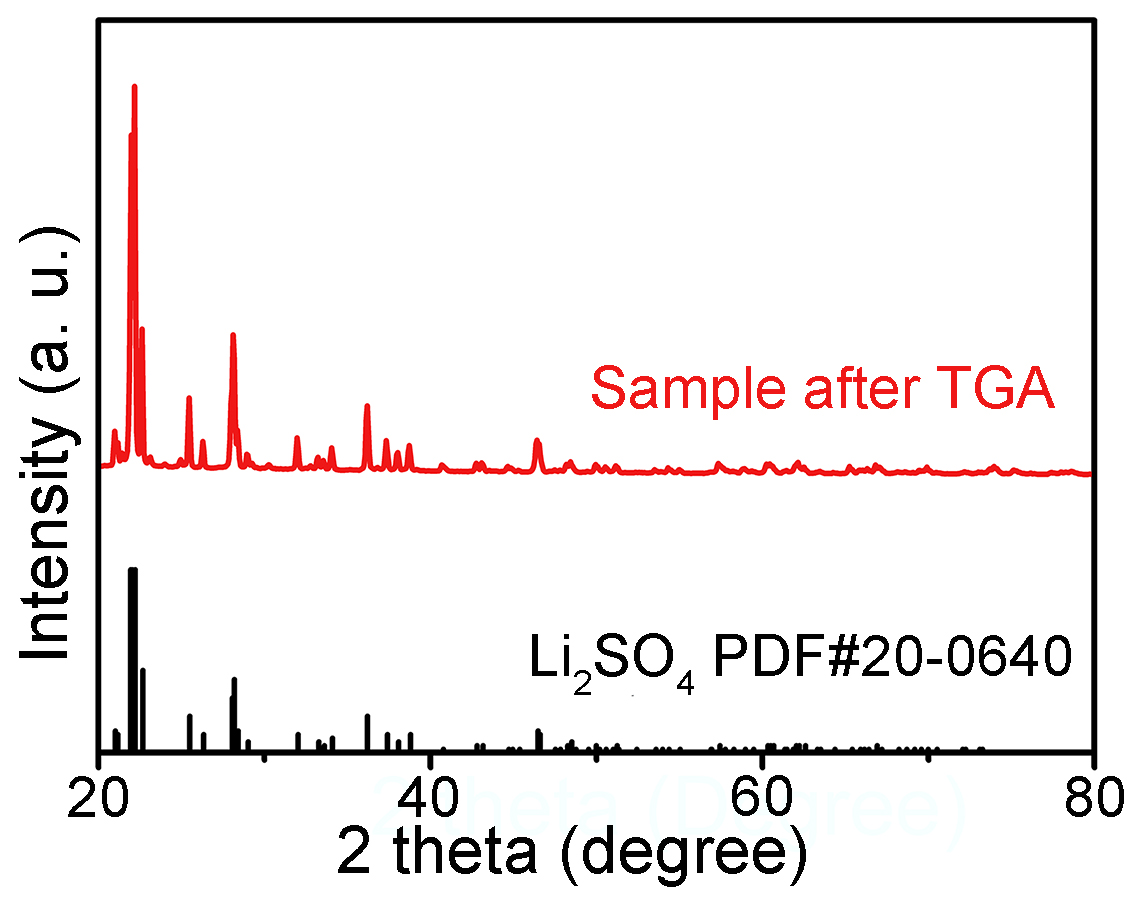


**Figure S10**. XRD of the residues after TGA measurement in air of the Li_2_S@P-CNFs nanofibers.





**Figure S11**. XPS spectra of the Li 1*s* of the Li_2_S@P-CNFs nanofibers.


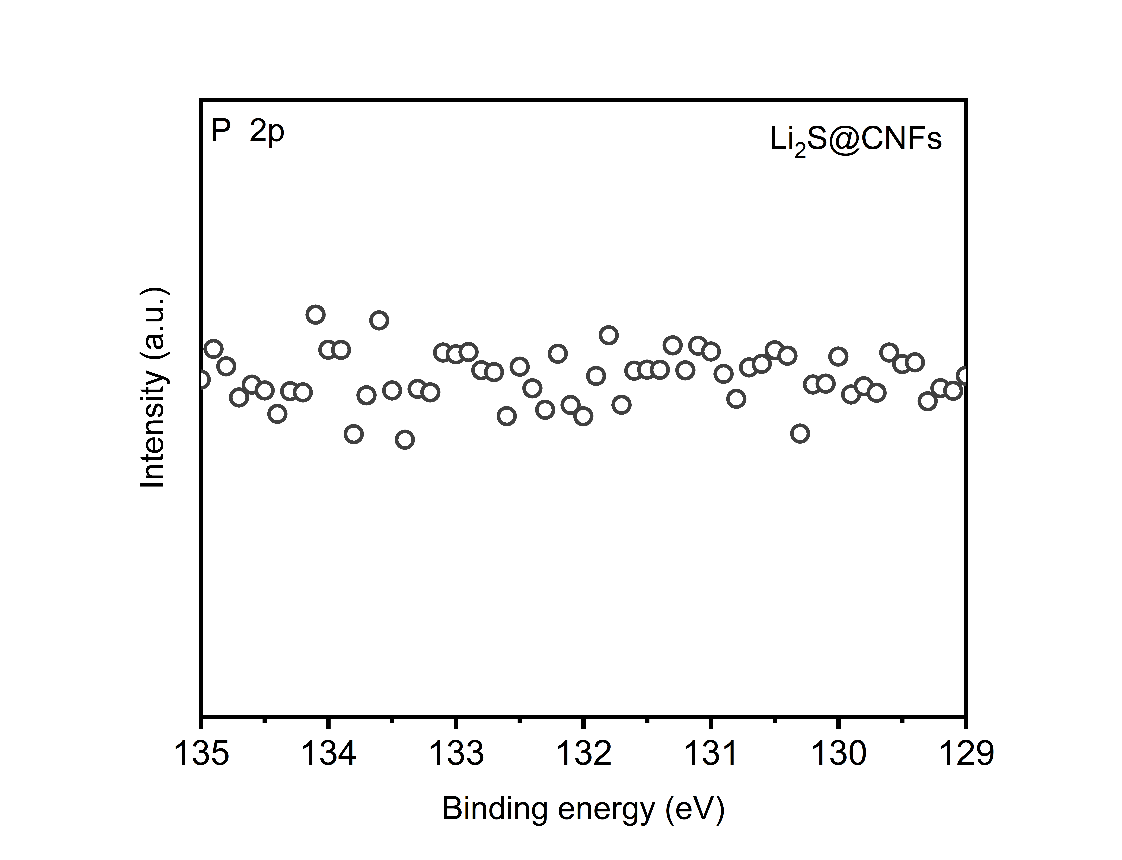


**Figure S12**. XPS spectra of the P 2*p* of the Li_2_S@CNFs nanofibers.


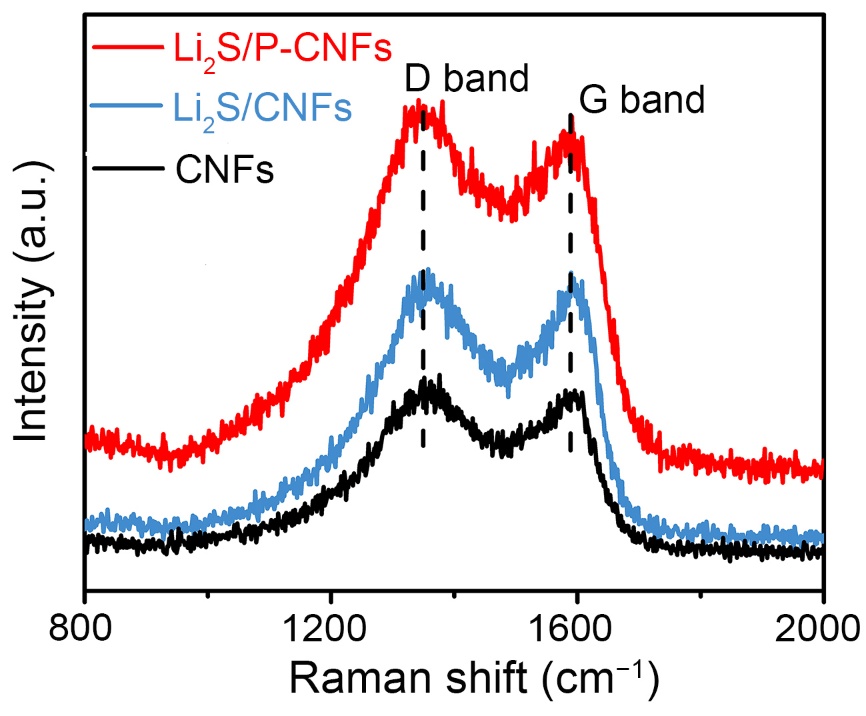


**Figure S13**. Raman spectrum of the Li_2_S@P-CNFs, Li_2_S@CNFs, and CNFs in the wave number range of 800 to 2000 cm^−1^.


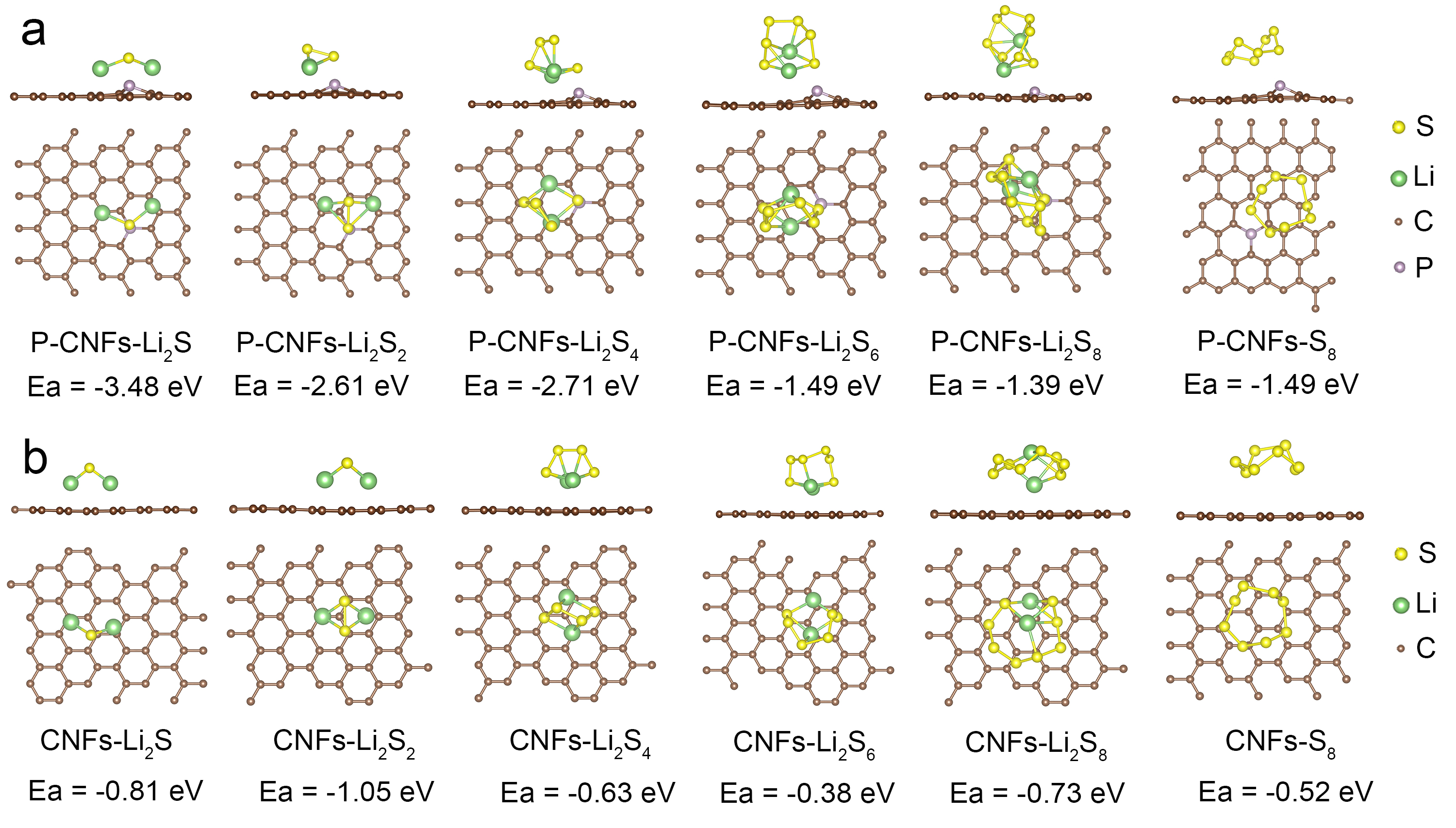


**Figure S14**. Optimized configurations for the binding of representative LiPSs species (Li_2_S, Li_2_S_2_, Li_2_S_4_, Li_2_S_6_, Li_2_S_8_, and S_8_) to (a) P-CNFs and (b) CNFs.


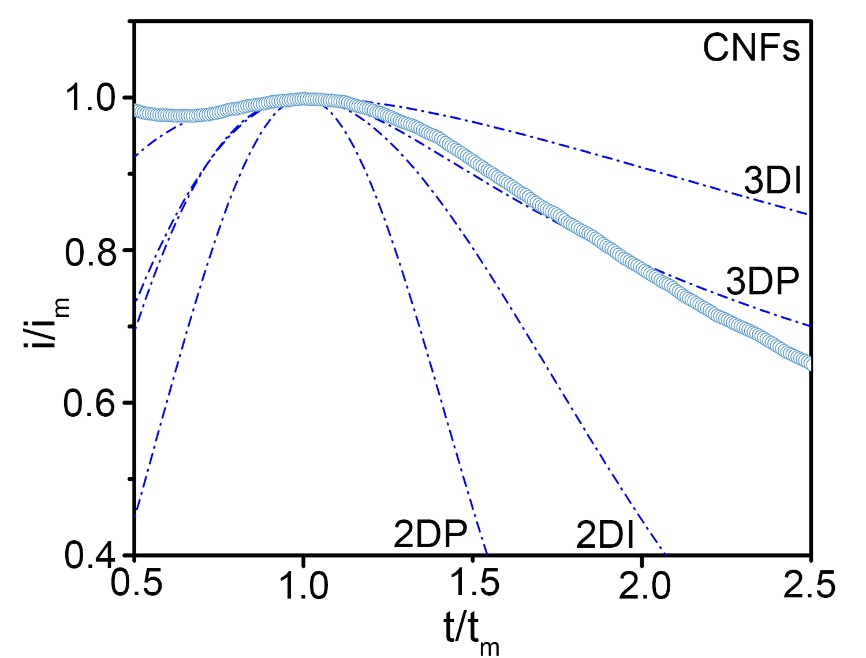


**Figure S15**. Corresponding dimensionless transients of CNFs compared with theoretical growth models. (t: time, t_m_: time needed to reach the maximum current; i: current, i_m_: maximum current).


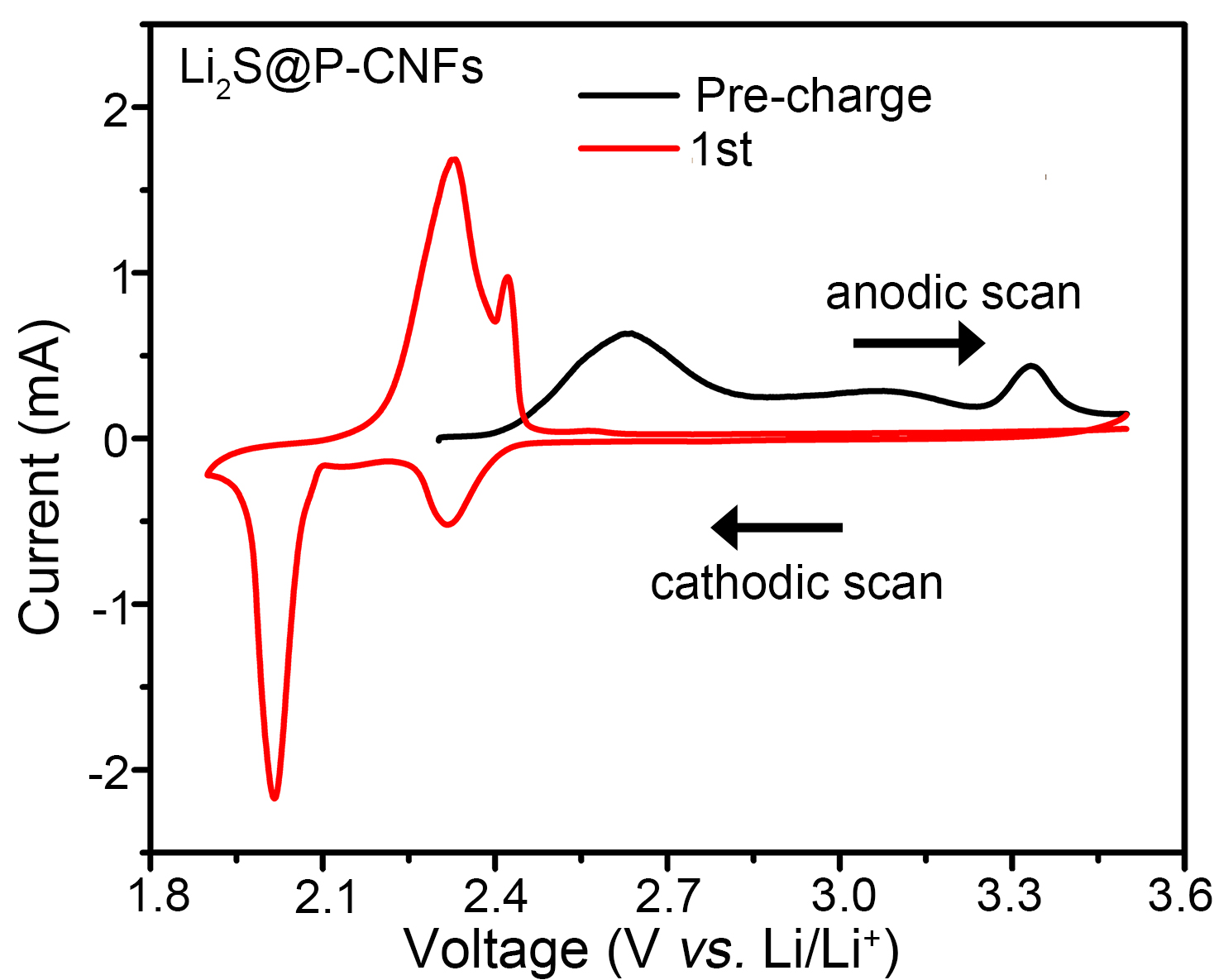


**Figure S16**. Cyclic voltammetry of the coin cell with the Li_2_S@P-CNFs electrode in the voltage range of 1.9-3.5 V during the initial activation process and first cycle at a scan rate of 0.1 mV s^−1^.


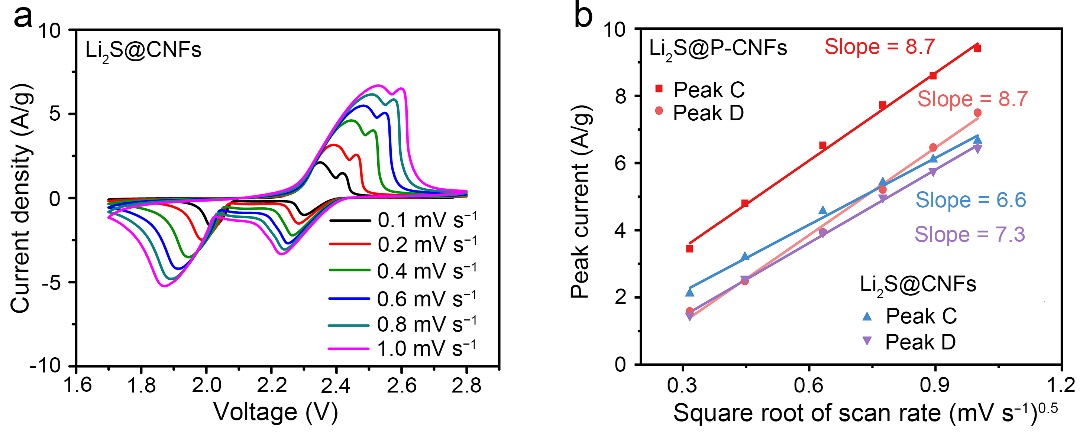


**Figure S17**. (a) Cyclic voltammetry of the coin cell with the Li_2_S@CNFs electrode in the voltage range of 1.7-2.8 V at various scan rates of 0.1, 0.2, 0.4, 0.6, 0.8, and 1.0 mV s^−1^. (b) The plot of CV peak current of the peak C (Li_2_S→Li_2_S_4_) and the peak D (Li_2_S_4_→S_8_) versus the square root of scan rate.


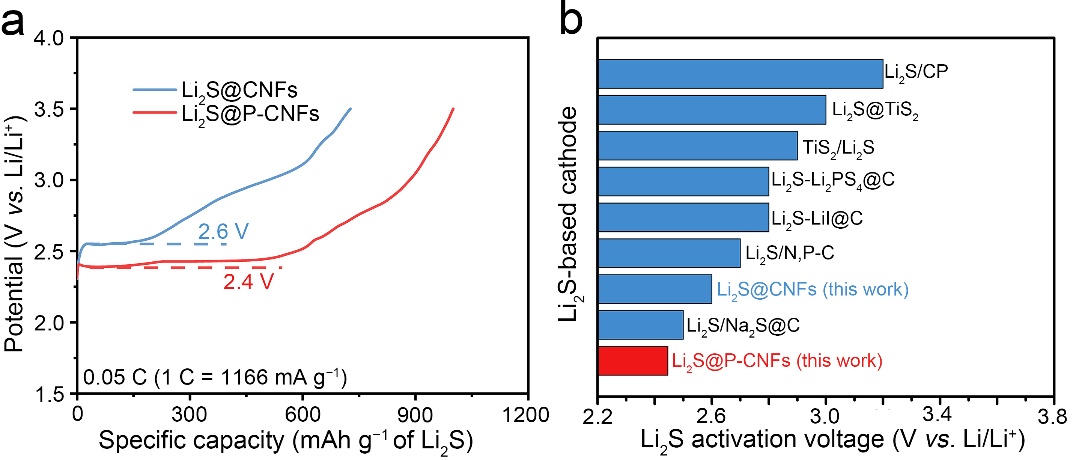


**Figure S18**. (a) Pre-charging curve of the Li-S coin cells based on Li_2_S@P-CNFs and Li_2_S@CNFs electrode at a current density of 0.05 C. (b) A comparison of Li_2_S@P-CNFs and reported Li_2_S-based cathodes in the activation potential barrier. (Li_2_S/Na_2_S@C,^[5]^ Li_2_S/N,P-C,^[6]^ Li_2_S-LiI@C,^[7]^ Li_2_S-Li_2_PS_4_@C,^[8]^ TiS_2_/Li_2_S,^[9]^ Li_2_S@TiS_2_,^[10]^ Li_2_S/CP^[11]^)


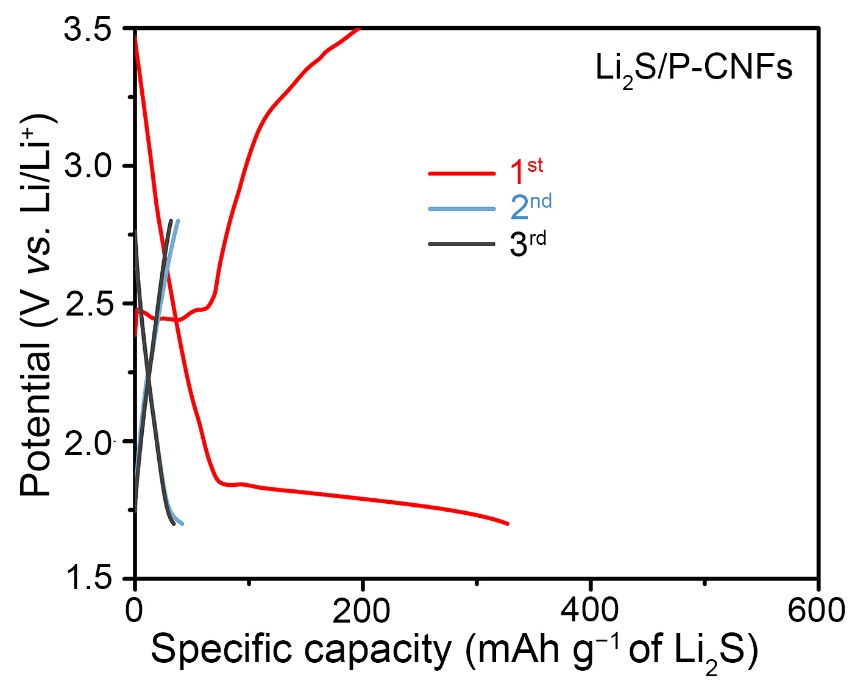


**Figure S19**. The first three cycles of the Li_2_S/P-CNFs at 0.05 C. The Li_2_S/P-CNFs were prepared by soaking the P-CNFs in 5 mM Li_2_S solution (solvent: ethanol) in a glove box and naturally dried. The areal loading of Li_2_S in Li_2_S/P-CNFs is around 1 mg cm^−2^. The coin cell assembly and testing are consistent with the Li_2_S@P-CNFs electrodes.


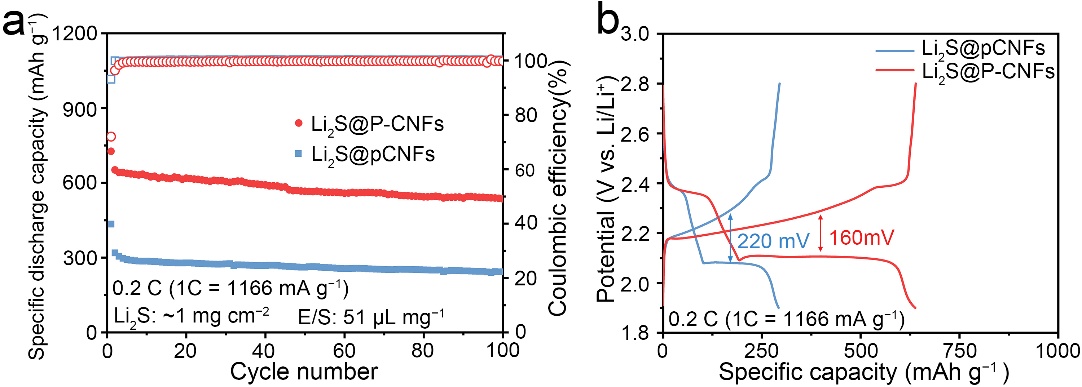


**Figure S20**. (a) The specific discharge capacity of the Li-S coin cells based on Li_2_S@P-CNFs or Li_2_S@CNFs cathodes at a current density of 0.2 C. (b) Galvanostatic charge-discharge (GCD) curves of the coin cells based on Li_2_S@P-CNFs and Li_2_S@CNFs.


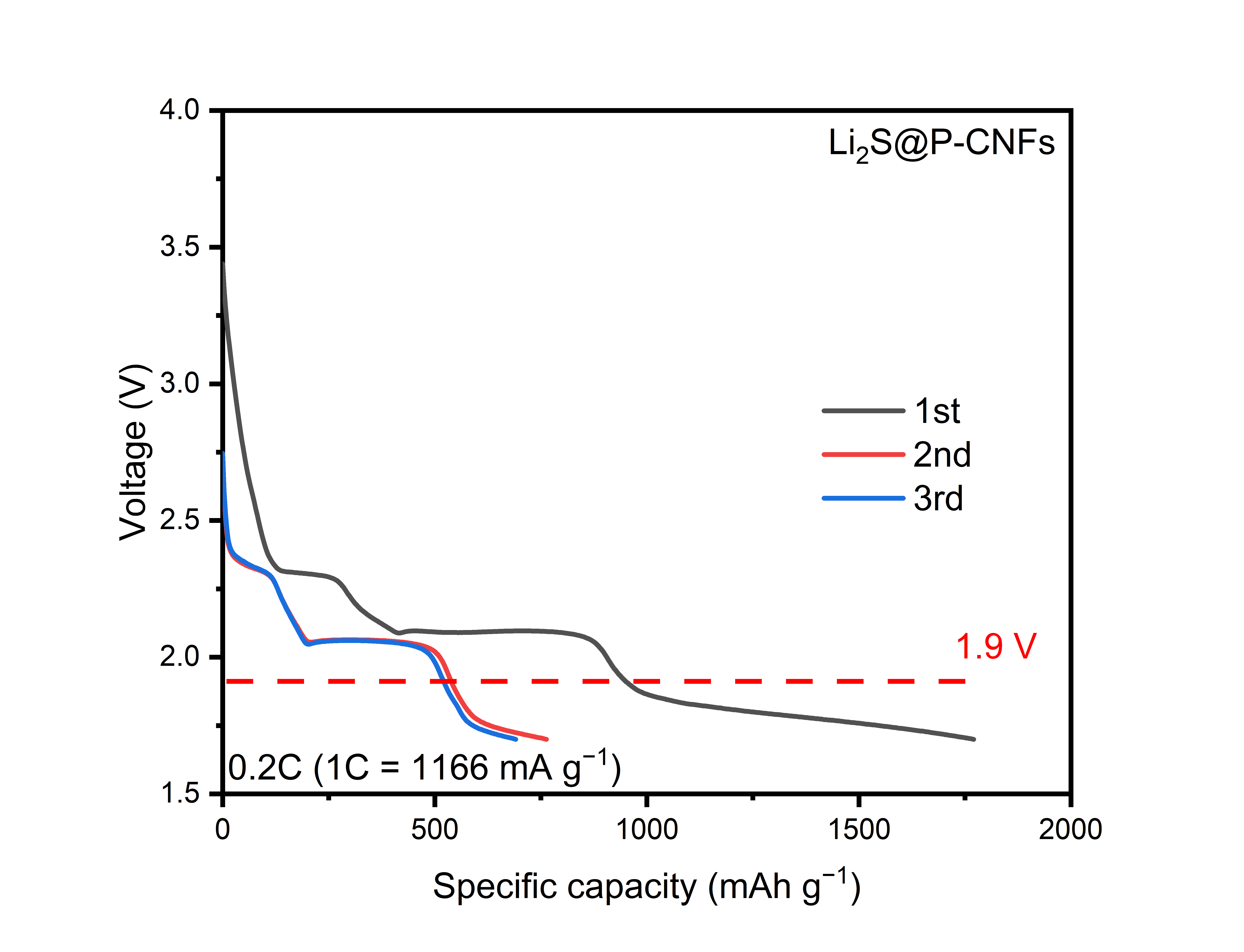


**Figure S21**. Discharge curves of Li_2_S@P-CNFs at cut-off voltages of 1.7 V.

*
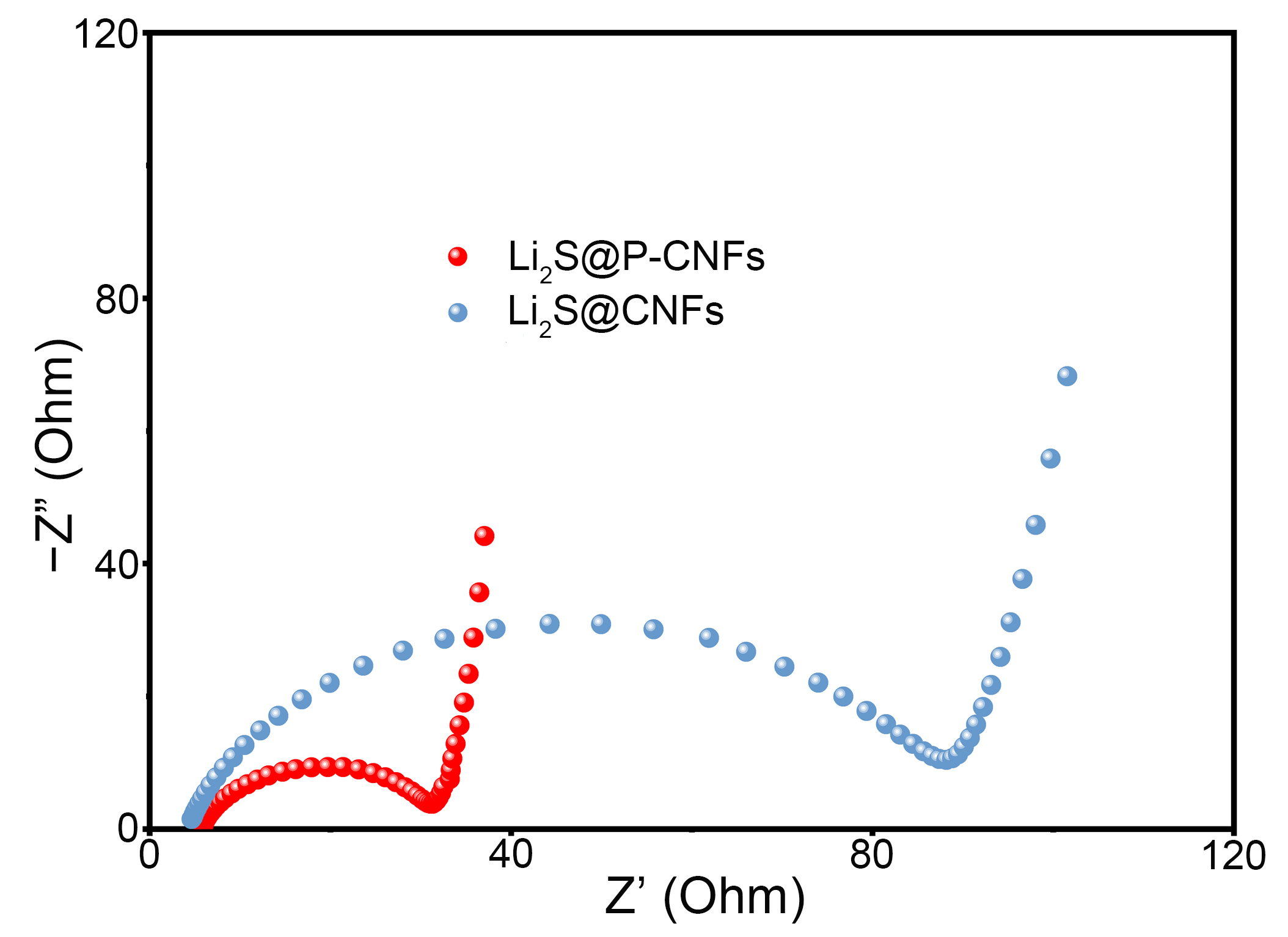
*

**Figure S22**. Electrochemical Impedance Spectroscopy of the Li−S batteries with the Li_2_S@P-CNFs and Li_2_S@CNFs as the electrode.


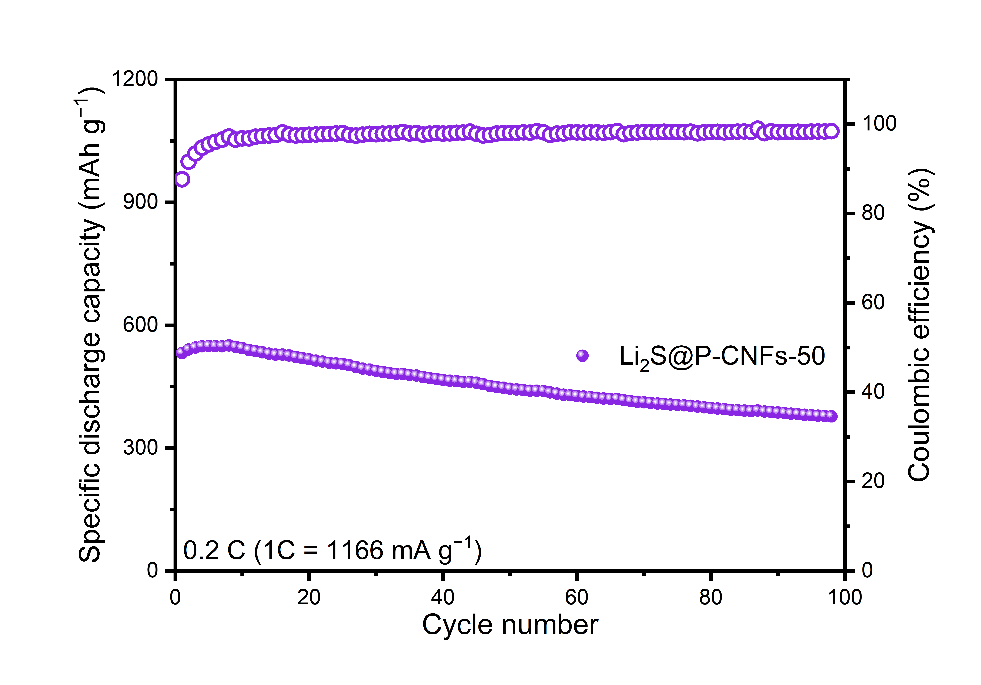


**Figure S23**. The specific discharge capacity of the Li−S coin cells based on Li_2_S@P-CNFs-50 cathodes at a current density of 0.2 C. The coin cells were assembled with Li_2_S@P-CNFs-50 as the cathode and lithium foil as the anode. The Li_2_S loading in the cathodes was approximately 1.0 mg cm^−2^.


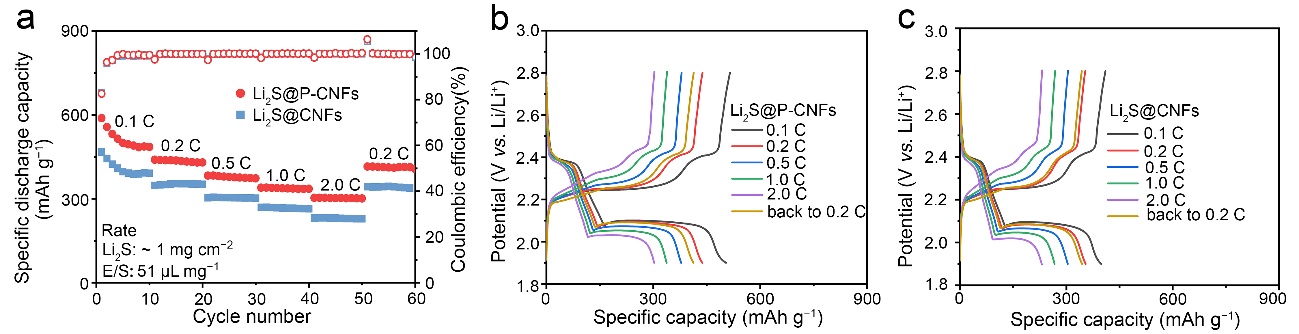


**Figure S24**. (a) Rate performance of the Li−S coin cells based on Li_2_S@P-CNFs and Li_2_S@CNFs cathodes. GCD curves of the coin cells based on (b) Li_2_S@P-CNFs and (c) Li_2_S@CNFs cathodes at different current densities.


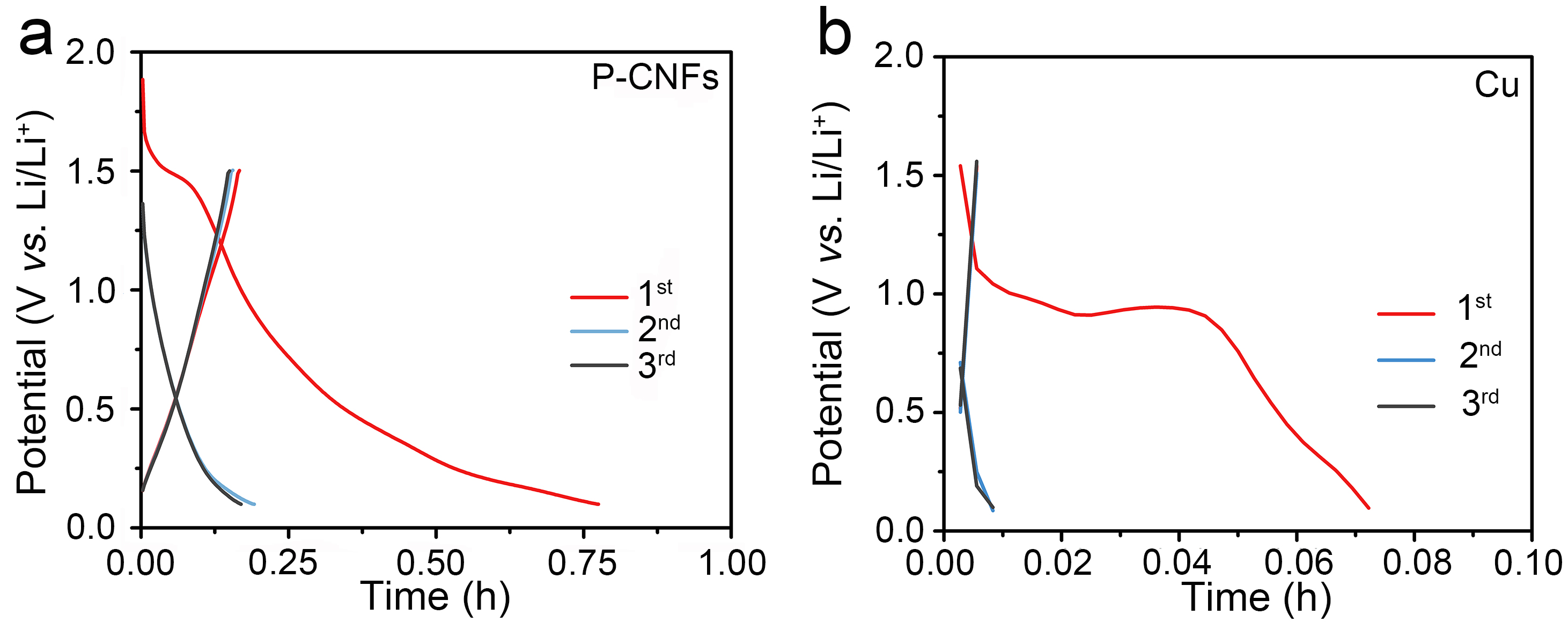


**Figure S25**. The first three cycles of the (a) Li||PCNFs and (b) Li||Cu cells at a current density of 0.1 mA cm^−2^ from 0.1 V to 2.0 V.


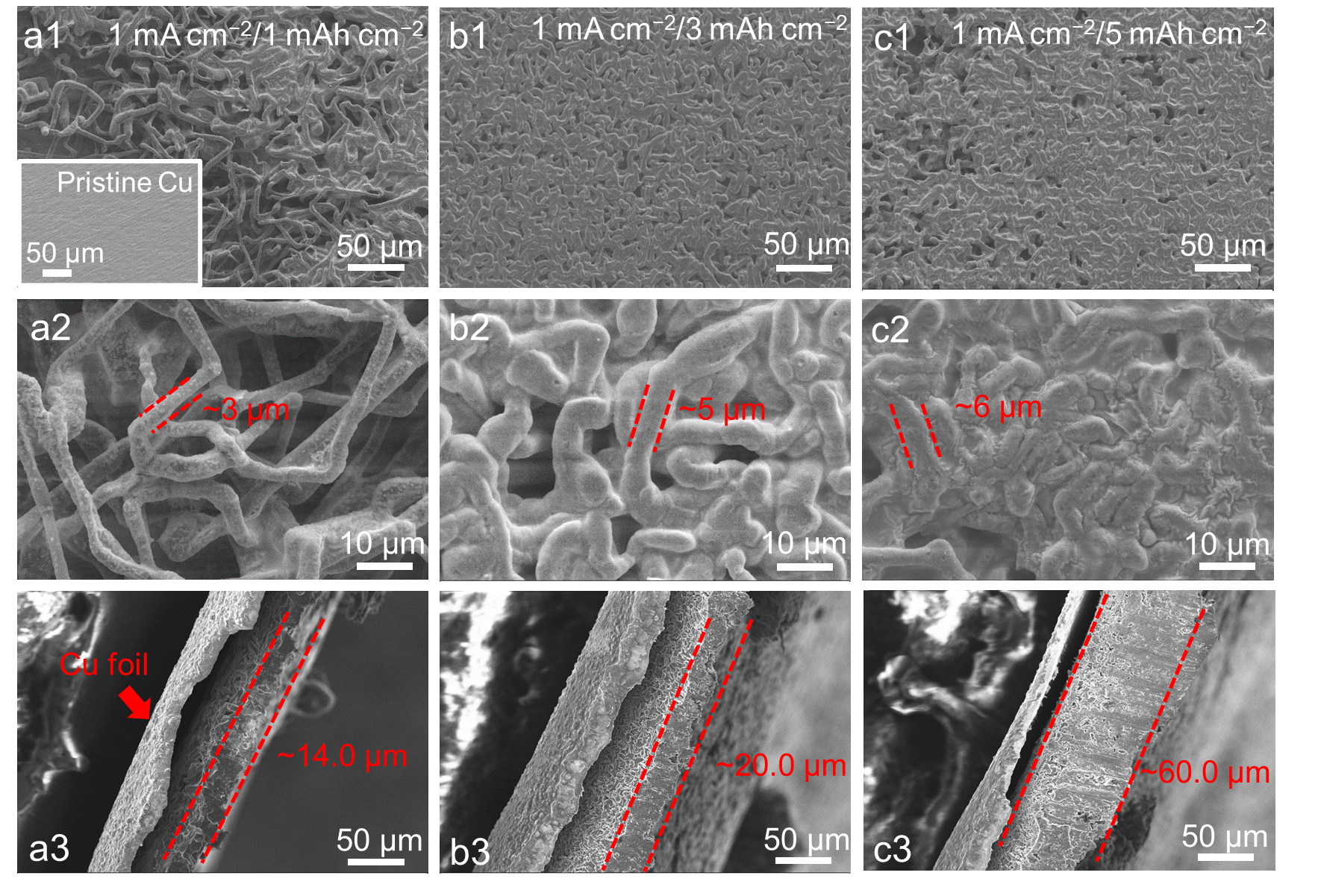


**Figure S26**. The micro-morphology evolution of Li during plating onto Cu current collector at a current density of 1 mA cm^−2^ with various areal capacities of (a) 1 mAh cm^−2^, (b) 3 mAh cm^−2^, and (c) 5 mAh cm^−2^ of Li.


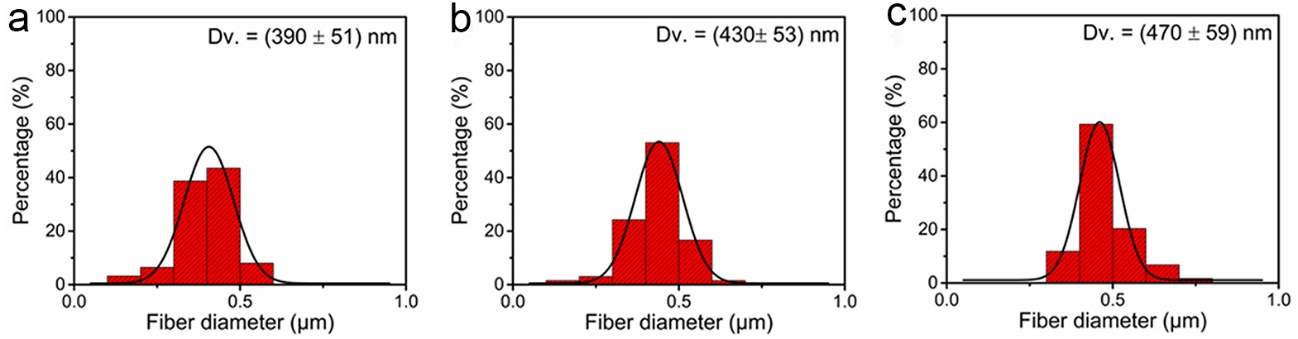


**Figure S27**. Diagram of statistical analysis of the average diameter for P-CNFs after (a) 1, (b) 3, and (c) 5 mAh cm^−2^ Li deposition at 1 mA cm^−2^ from their SEM images.


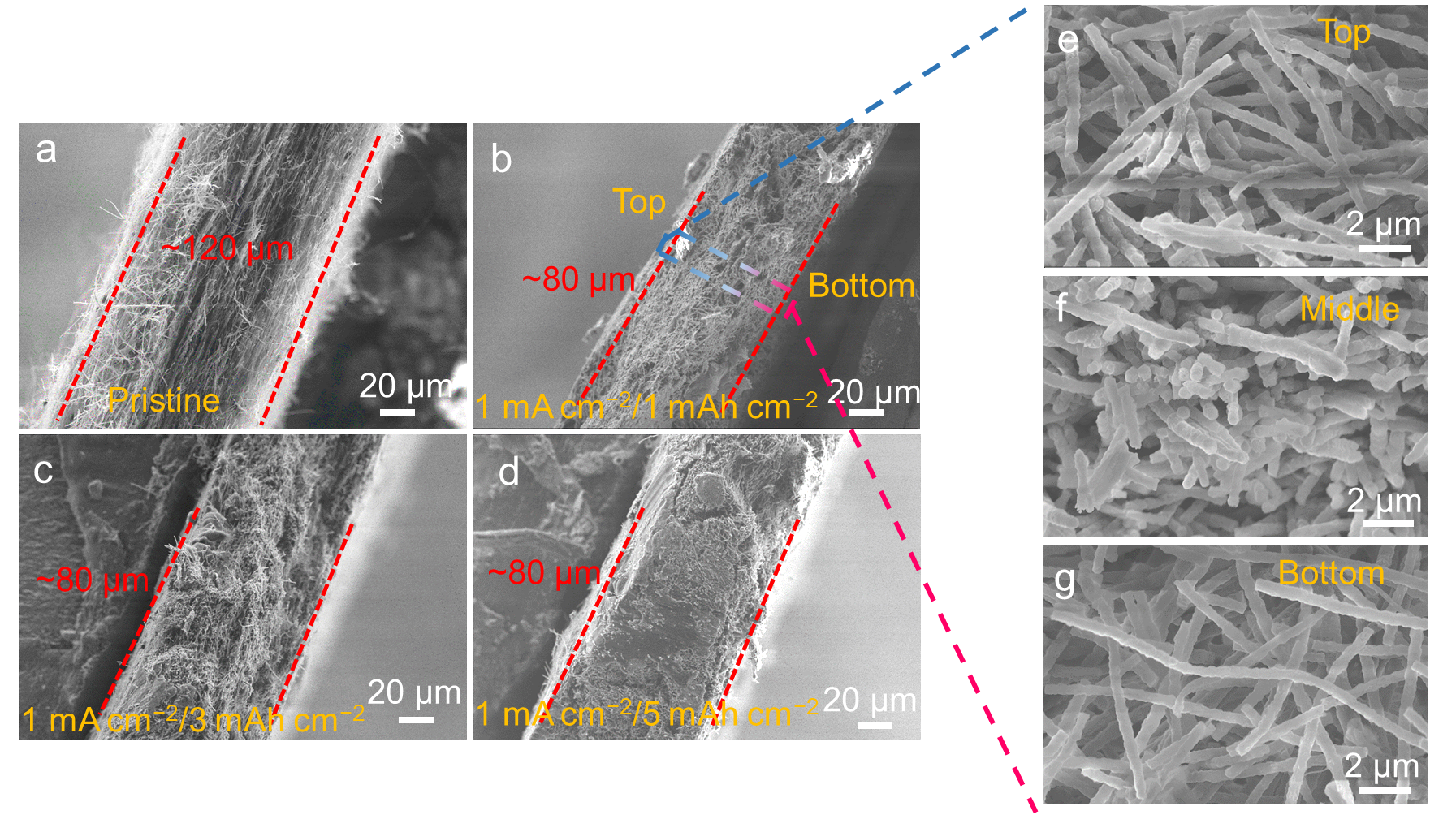


**Figure S28**. The cross-section of the (a) pristine P-CNFs, after (b) 1, (c) 3, and (d) 5 mAh cm^−2^ Li deposition. The enlarged SEM images of the P-CNFs with 1 mAh cm^−2^ Li deposition at (e) top, (f) middle, and (g) bottom.


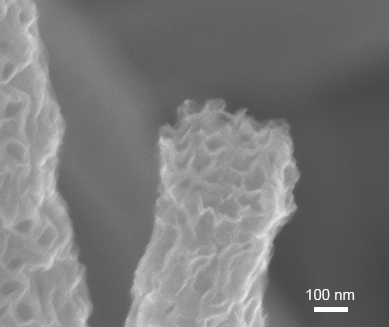


**Figure S29**. SEM image of the pristine P-CNFs current collector.


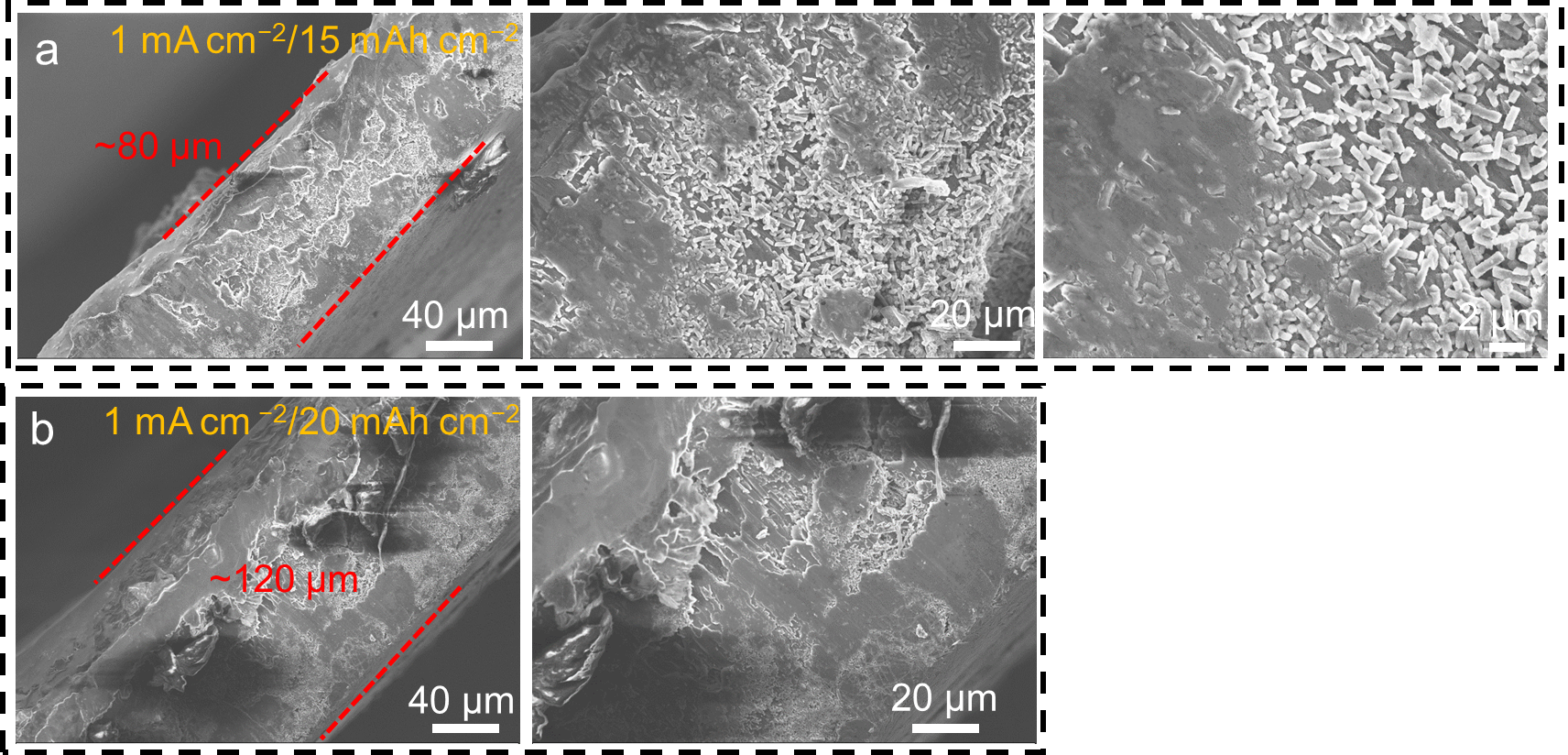


**Figure S30.** The cross-section SEM images of the P-CNFs current collector, after (a) 15 and (b) 20 mAh cm^−2^ Li deposition.


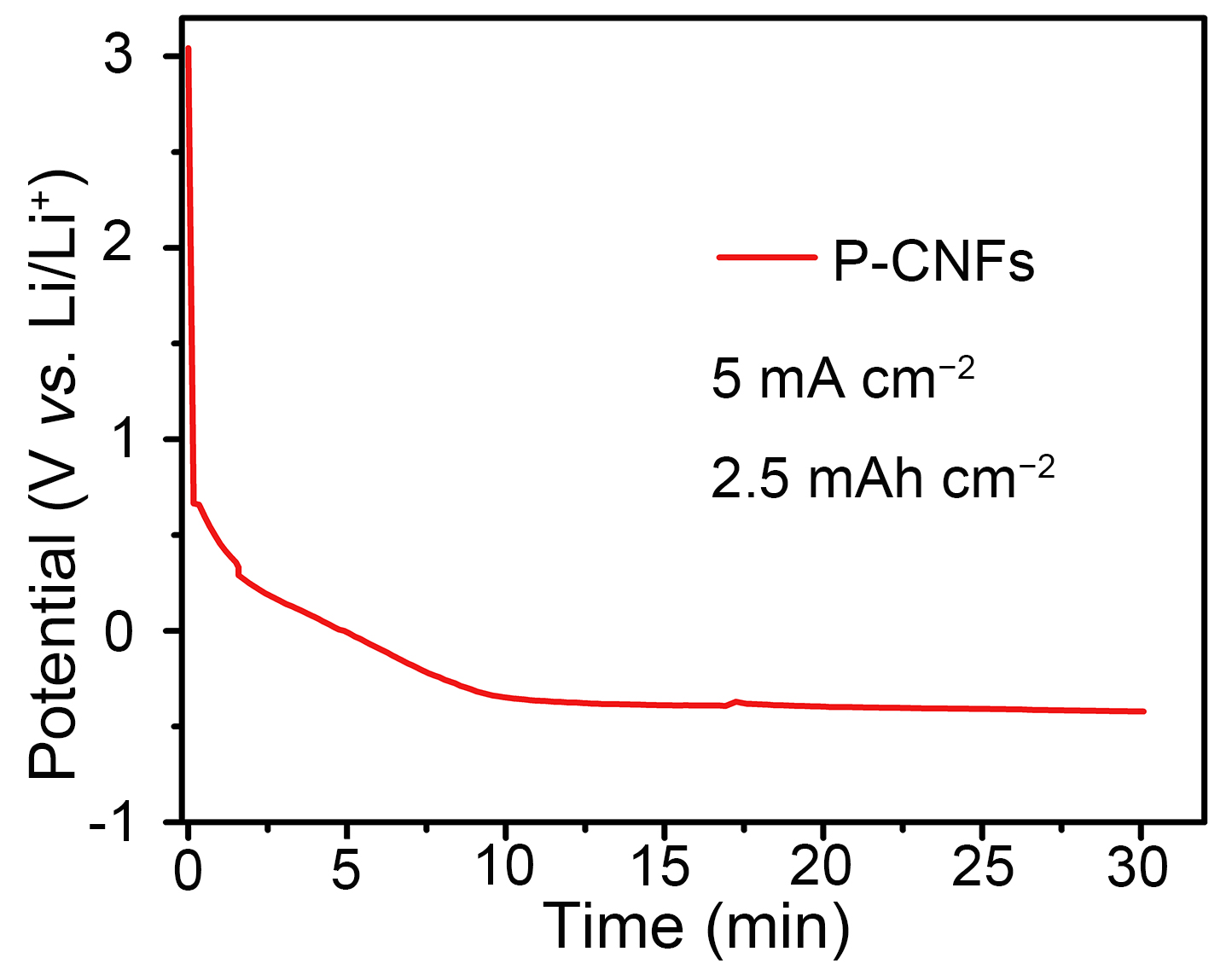


**Figure S31**. The discharge curve of the *in-situ* optical Li||P-CNFs cell at a current density of 5 mA cm^−2^.


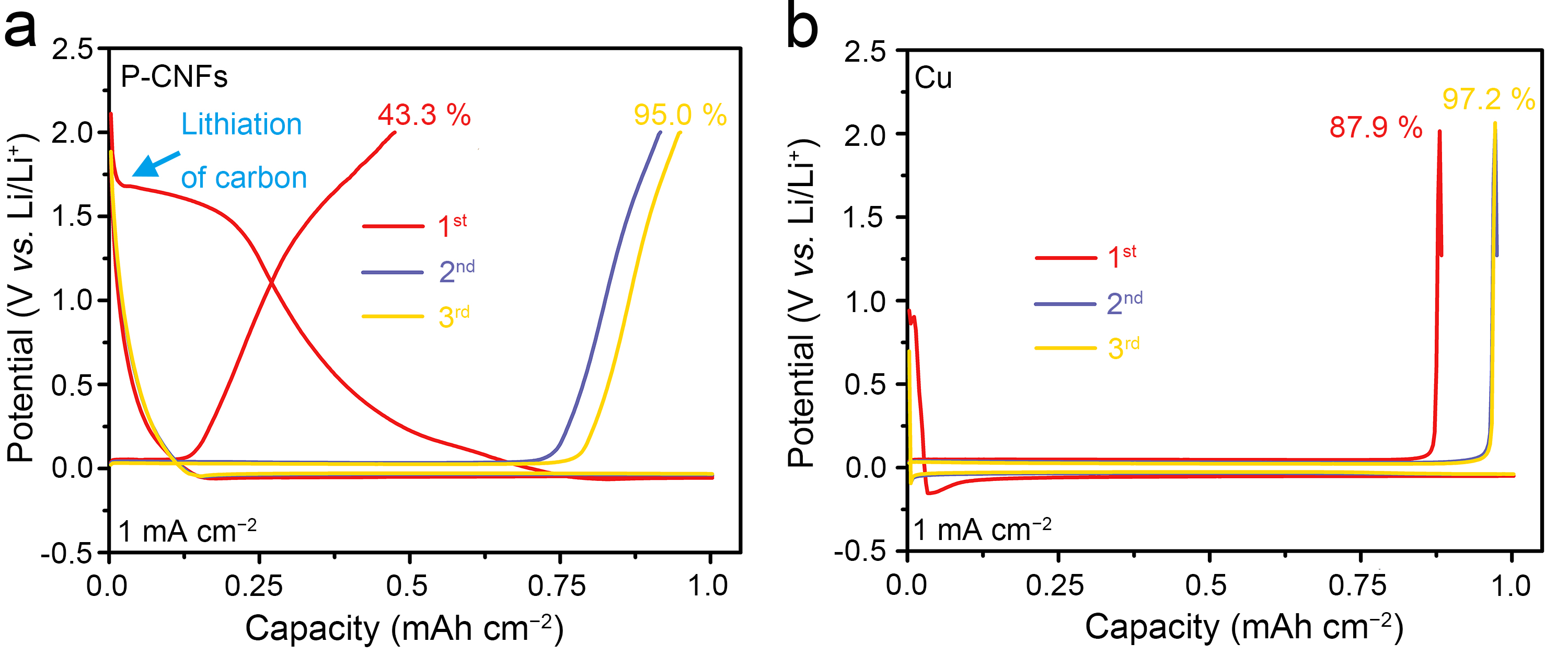


**Figure S32**. The initial three Li plating and stripping processes on (a) P-CNFs and (b) Cu current collectors.


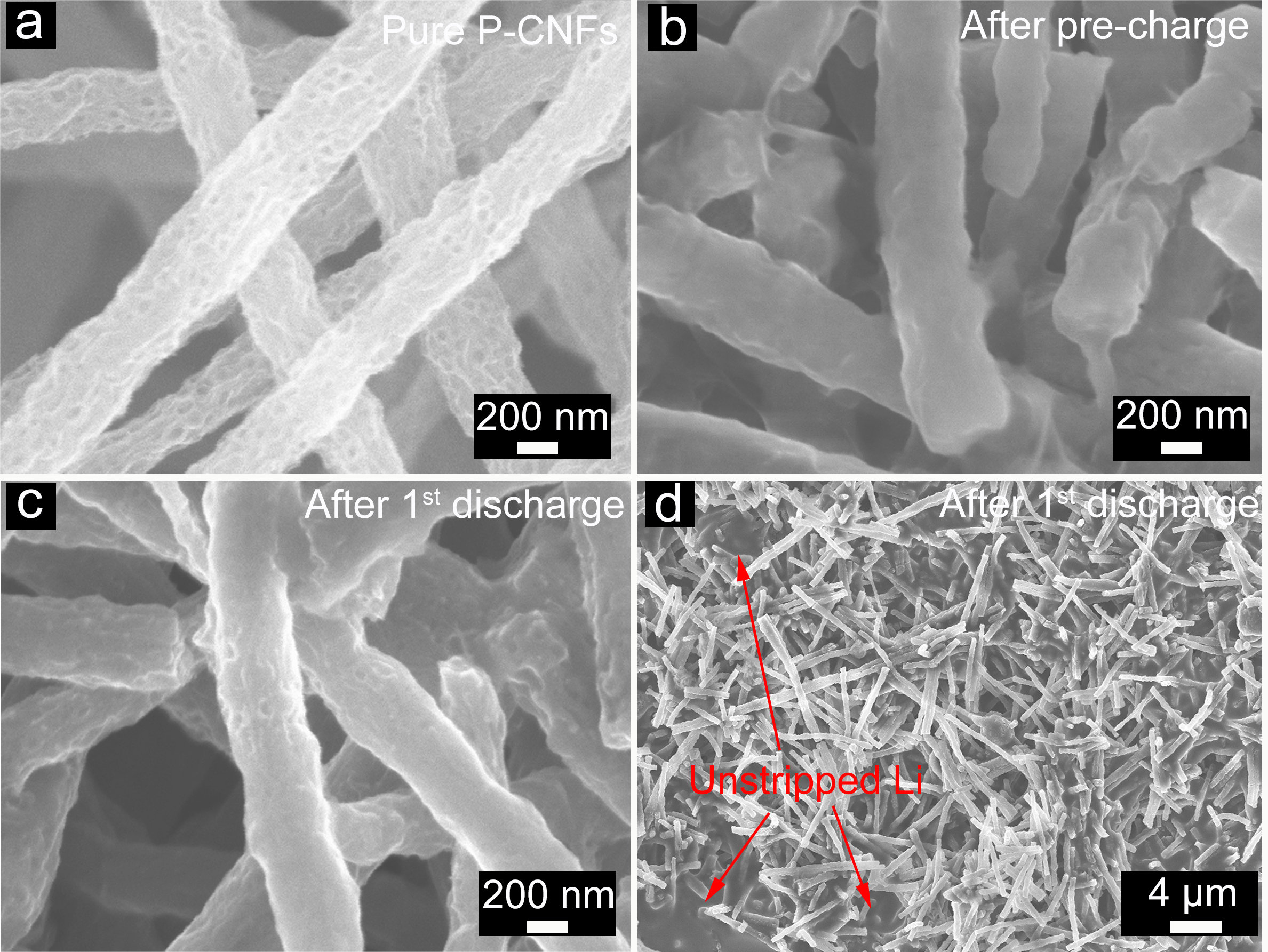


**Figure S33**. The SEM images of the (a) pure P-CNFs, (b) the P-CNFs anode after the pre-charge, and (c, d) the P-CNFs anode after the first discharge of the Li_2_S@P-CNFs||P-CNFs full cell.


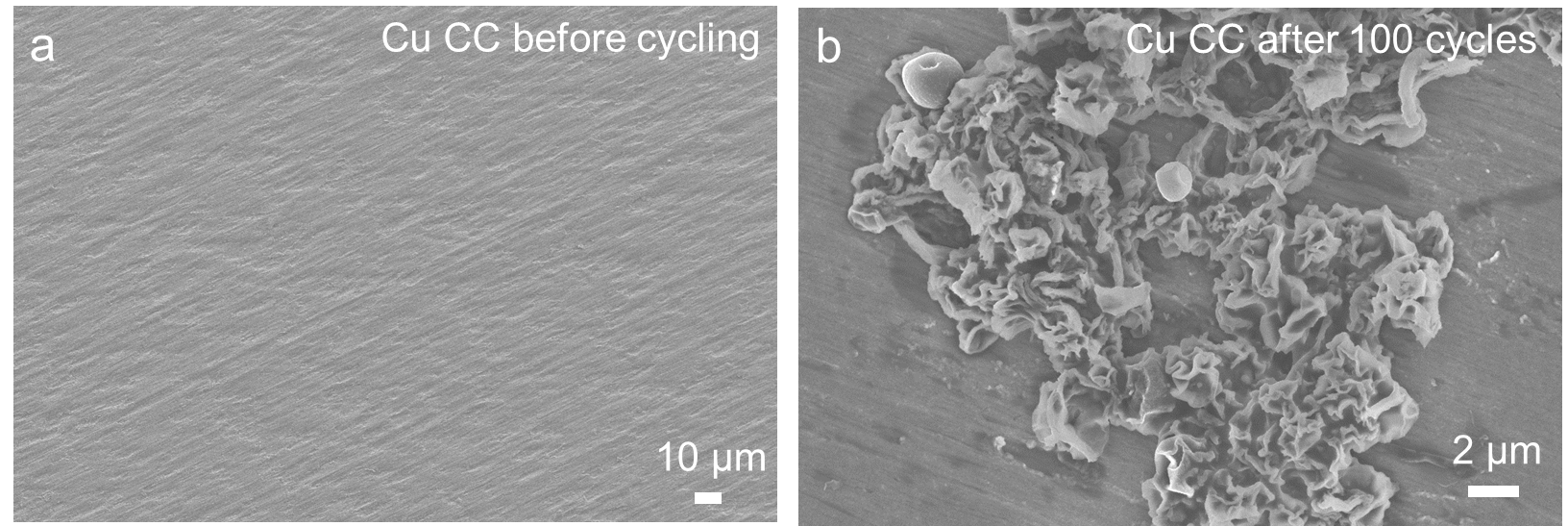


**Figure S34**. SEM images of the (a) pristine Cu current collectors (CC) and (b) Cu CC taken from Li_2_S@P-CNFs||Cu full cells after 100 cycles.


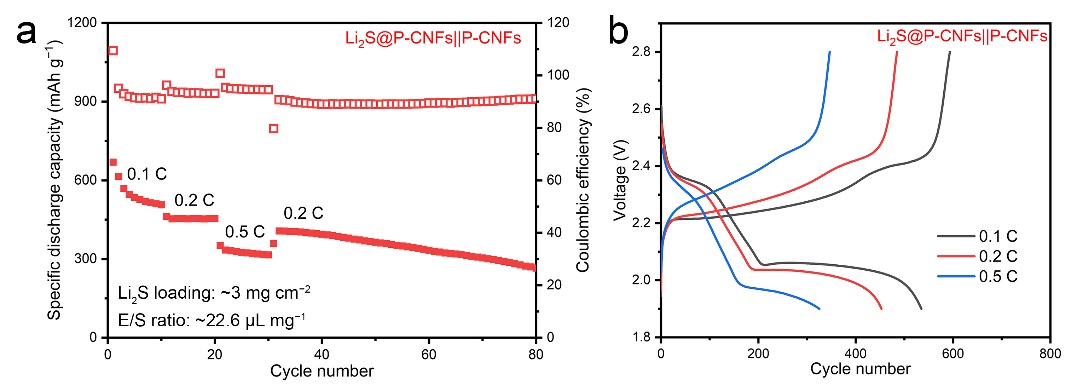


**Figure S35**. (**a**) Specific discharge capacity of the Li_2_S@P-CNFs||P-CNFs full cells at 0.1 C, 0.2 C, 0.5 C, and back to 0.2 C. The Li_2_S loading is 3 mg cm^−2^ and electrolyte to sulfur (E/S) ratio is 22.6 μL mg^−1^. (**b**) GCD curves of the Li_2_S@P-CNFs||P-CNFs at 0.1 C, 0.2 C, and 0.5 C.

**Calculation of the theoretical thickness of Li metal**

**Equation S3**:

*T*$=\frac{QM}{zF} \times\frac{1}{\rho}$ S3

Where *T* is the thickness, *Q* is the areal capacity (mA h cm^−2^), *M* is the molar mass of Li (6.94 g mol^−1^), *z* is the number of electrons transferred during Li plating (in this case, *z* = 1), *F* is the Faraday constant (96485 C mol^−1^), *ρ* is the density of Li metal (0.534 g cm^−3^). Accordingly, it can be calculated that a Li deposition capacity of 1, 3, and 5 mA h cm^−2^ corresponds to a thickness of 4.8, 14.4, and 24.2 µm, respectively. For example, when the deposition capacity is 5 mA h cm^−2^, the corresponding thickness can be calculated as follows:

*T*$=\frac{5 mAh {cm}^{-2} \times6.94 g {mol}^{-1}}{96485 C{mol}^{-1}} \times\frac{1}{0.534 g {cm}^{-3}}$

$$=0.00242 cm=24.2 \mu m$$

**Calculation of the energy density of the full cell**

**Equation S4**:

*E*$=\frac{C_{cell} V_{avg}}{m_{total}}$ S4

Where *E* is the energy density of the full cell (Wh kg^−1^), *C_cell_* is the capacity of the full cell (mAh), *V_avg_* is average discharge voltage (2.1 V), *m_total_* is the total mass of the electrode (mg). In our case, we use the discharge capacity at 0.1 C in **Figure S35** for calculating the energy density of the full cell. The corresponding energy density can be calculated as follows:

*E*$=\frac{2.01 mAh \times2.1 V}{\left( 7+2 \right)\times0.001}=$*469* Wh kg^−1^

**Table S1**. Detailed parameters and calculated Li diffusion coefficient (𝐷_𝐿𝑖_) values at different redox peak positions for different electrodes. The number of electrons transferred (n), the surface area of the electrode (A), and the concentration of Li^+^ ions in the cathodes are 2, 1.27 cm^−2^, and 0.0012 mol cm^−3^, respectively.

| Electrodes | Peak | Slope, *Ip / v*^0.5^ | $D_{Li}$ / cm^2^ s^−1^ |
| --- | --- | --- | --- |
| Li_2_S@P-CNFs | A | 0.123 | 1.1 × 10^−8^ |
|  | B | 0.196 | 2.9 × 10^−8^ |
|  | C | 0.275 | 5.6× 10^−8^ |
|  | D | 0.275 | 5.6× 10^−8^ |
| Li_2_S@ CNFs | A | 0.108 | 8.7 × 10^−9^ |
|  | B | 0.161 | 1.9 × 10^−8^ |
|  | C | 0.209 | 3.2 × 10^−8^ |
|  | D | 0.231 | 4.0 × 10^−8^ |

**Table S2**. Comparison of the electrochemical performance of different Li-free Li−S batteries based on Li_2_S cathodes.

| Cathode | Anode | Self-supporting | Li_2_S loading (mg cm^−2^) | Electrolyte to sulfur ratio (μL mg^−1^) | Current density (C) | Initial capacity  (mAh g^−1^) | Final capacity (mAh g^−1^) | Cycle number | Ref. |
| --- | --- | --- | --- | --- | --- | --- | --- | --- | --- |
| Li_2_S@P-CNFs | P-CNFs | Yes | ~1.0 | ~68 | 0.1 | 785.7 | 361.4 | 100 | This work |
|  |  |  |  |  | 1.0 | 654.6 | 261.5 | 1000 |  |
|  |  |  | 3.0 | 22.6 | 0.1~0.5 | 527.3 (at 0.1C) | 265.4 (at 0.2C) | 80 |  |
| Li_2_S | SnO_2_ | Yes | 2.4 | 24.0 | 0.5 | ~740.0 | 647.0 | 200 | 12 |
| Li_2_S | Fe_3_O_4_/CNs | Yes | 3.0 | 28.7 | 0.2 | 576.0 | 345.6 | 50 | 13 |
| Li_2_S | Au/Cu | Yes | 4.0 | 17.1 | 0.1 | 639.0 | 409.0 | 50 | 14 |
| Li_2_S/C | Sn | No | - | Gel electrolyte | 0.2 | ~210.0 | 150.0 | 90 | 15 |
| Li_2_S@MX | Cu | No | 5 | Gel electrolyte | 0.2 | 819.0 | 655.2 | 300 | 16 |
| Li_2_S@graphene | Graphite | No | 2.0 | 12.5 | 0.14 | ~720.0 | 440.0 | 200 | 17 |
| Li_2_S-ZnS@NC | Si-NP@HCF | No | 2−7 | 17.1 | 0.2 | 710 | 409.7 | 200 | 18 |

**References**

[1] Y. Lu, M. Ballauff, *Prog. Polym. Sci.* **2016**, *59*, 86-104.

[2] a) T. L. Zheng, J. W. Xiong, X. T. Shi, B. Y. Zhu, Y. J. Cheng, H. B. Zhao, Y. G. Xia, *Energy Storage Mater.* **2021**, *38*, 599-608; b) Z. C. Wang, Y. Y. Sun, Y. Y. Mao, F. R. Zhang, L. Zheng, D. S. Fu, Y. B. Shen, J. C. Hu, H. L. Dong, J. J. Xu, X. D. Wu, *Energy Storage Mater.* **2020**, *30*, 228-237.

[3] a) T. L. Zheng, J. W. Xiong, B. Y. Zhu, X. T. Shi, Y. J. Cheng, H. B. Zhao, Y. G. Xia, *J. Mater. Chem. A* **2021**, *9*, 9307-9318; b) X. Q. Zhang, X. Chen, X. B. Cheng, B. Q. Li, X. Shen, C. Yan, J. Q. Huang, Q. Zhang, *Angew. Chem. Int. Ed.* **2018**, *57*, 5301-5305.

[4] P. Feng, Q. P. Wu, Y. Rodriguez Ayllon, Y. Lu, *Chem. -Eur. J.* **2024**, *30*, e202401345.

[5] M. Li, J. Lu, J. Y. Shi, S. B. Son, D. Luo, I. Bloom, Z. W. Chen, K. Amine, *J. Am. Chem. Soc.* **2021**, *143*, 2185-2189.

[6] J. Zhang, Y. Shi, Y. Ding, L. L. Peng, W. K. Zhang, G. H. Yu, *Adv. Energy Mater.* **2017**, *7*, 1602876.

[7] F. Wu, J. T. Lee, N. Nitta, H. Kim, O. Borodin, G. Yushin, *Adv. Mater.* **2015**, *27*, 101-108.

[8] M. Li, Z. Y. Bai, Y. J. Li, L. Ma, A. Dai, X. F. Wang, D. Luo, T. P. Wu, P. Liu, L. Yang, K. Amine, Z. W. Chen, J. Lu, *Nat. Commun.* **2019**, *10*,1890.

[9] G. M. Zhou, H. Z. Tian, Y. Jin, X. Y. Tao, B. F. Liu, R. F. Zhang, Z. W. She, D. Zhou, Y. Y. Liu, J. Sun, J. Zhao, C. X. Zu, D. S. Wu, Q. F. Zhang, Y. Cui, *Proc. Natl. Acad. Sci*. *USA*, **2017**, *114*, 840-845.

[10] Z. W. Seh, J. H. Yu, W. Li, P.-C. Hsu, H. Wang, Y. Sun, H. Yao, Q. Zhang, Y. Cui, *Nat. Commun.* **2014**, *5*, 5017.

[11] Q. N. Fang, J. C. jia, S. L. Zhang, T. F. Zhou, W. K. Pang, Q. F. Gu, H. K. Liu, Z. P. Guo, J. Z. Wang, *Adv. Energy Mater.* **2021**, *11*, 2100957.

[12] M. Liu, Y. X. Ren, H. R. Jiang, C. Luo, F. Y. Kang, T. S. Zhao, *Nano Energy* **2017**, *40*, 240-247.

[13] M. L. Yu, Z.Y. Wang, Y. W. Wang, Y. F. Dong, J. S. Qiu, *Adv. Energy Mater.* **2017**, *7*, 1700018.

[14] J. Chen, J. W. Xiang, X. Chen, L. X. Yuan, Z. Li, Y. H. Huang, *Energy Storage Mater.* **2020**, *30*, 179-186.

[15] J. Hassoun, B. Scrosati, *Angew. Chem. Int. Ed.* **2010**, *49*, 2371-2374.

[16] Y. Z. Liu, X. Y. Meng, Z. Y. Wang, J. S. Qiu, *Nat. Commun.* **2022**, *13*, 4415.

[17] G. Q. Tan, R. Xu, Z. Y. Xing, Y. F. Yuan, J. Lu, J. G. Wen, C. Liu, L. Ma, C. Zhan, Q. Liu, T. P. Wu, Z. L. Jian, R. Shahbazian-Yassar, Y. Ren, D. J. Miller, L. A. Curtiss, X. L. Ji, K. Amine, *Nat. Energy* **2017**, *2*, 1-10.

[18] M. L. Yu, S. Zhou, Z. Y. Wang, W. Pei, X. J. Liu, C. Liu, C. L. Yan, X. Y. Meng, S. Wang, J. J. Zhao, J. S. Qiu, *Adv. Funct. Mater.* **2019**, *29*, 1905986.
